# Investigation of bacterial communities within the digestive organs of the hydrothermal vent shrimp *Rimicaris exoculata* provide insights into holobiont geographic clustering

**DOI:** 10.1371/journal.pone.0172543

**Published:** 2017-03-15

**Authors:** Dominique A. Cowart, Lucile Durand, Marie-Anne Cambon-Bonavita, Sophie Arnaud-Haond

**Affiliations:** 1 Ifremer (Institut Français de Recherche pour l'Exploitation de la MER) UMR MARBEC (Marine Biodiversity, Exploitation and Conservation) BP 17, Sète - France; 2 Ifremer (Institut Français de Recherche pour l'Exploitation de la MER) Laboratoire de Microbiologie des Environnements Extrêmes, UMR6197, Département des Ressources physiques et Ecosystèmes de Fond de mer (REM) ZI pointe du diable, CS 10070, Plouzané - France; 3 UBO, UMR 6197, UBO, Ifremer, CNRS, IUEM Rue Dumont d'Urville, Plouzané - France; 4 CNRS, UMR 6197, CNRS, Ifremer, UBO, IUEM Rue Dumont d'Urville, Plouzané - France; National Cheng Kung University, TAIWAN

## Abstract

Prokaryotic communities forming symbiotic relationships with the vent shrimp, *Rimicaris exoculata*, are well studied components of hydrothermal ecosystems at the Mid-Atlantic Ridge (MAR). Despite the tight link between host and symbiont, the observed lack of spatial genetic structure seen in *R*. *exoculata* contrasts with the geographic differentiation detected in specific bacterial ectosymbionts. The geographic clustering of bacterial lineages within a seemingly panmictic host suggests either the presence of finer scale restriction to gene flow not yet detected in the host, horizontal transmission (environmental selection) of its endosymbionts as a consequence of unique vent geochemistry, or vertically transmitted endosymbionts that exhibit genetic differentiation. To identify which hypothesis best fits, we tested whether bacterial assemblages exhibit differentiation across sites or host populations by performing a 16S rRNA metabarcoding survey on *R*. *exoculata* digestive prokaryote samples (n = 31) taken from three geochemically distinct vents across MAR: Rainbow, Trans-Atlantic Geotraverse (TAG) and Logatchev. Analysis of communities across two organs (digestive tract, stomach), three molt colors (white, red, black) and three life stages (eggs, juveniles, adults) also provided insights into symbiont transmission mode. Examining both whole communities and operational taxonomic units (OTUs) confirmed the presence of three main epibionts: *Epsilonproteobacteria*, *Mollicutes* and *Deferribacteres*. With these findings, we identified a clear pattern of geographic segregation by vent in OTUs assigned to *Epsilonproteobacteria*. Additionally, we detected evidence for differentiation among all communities associated to vents and life stages. Overall, results suggest a combination of environmental selection and vertical inheritance of some of the symbiotic lineages.

## Introduction

Deep-sea hydrothermal vent ecosystems are characterized by low oxygen, temperature extremes and toxic compounds, particularly during an eruption event [1]. Despite the harshness of these ecosystems, vents are home to a variety of specialized fauna that depend directly or indirectly upon chemosynthetic primary production [2], [3]. Bacterial communities serve as the primary producers at these ecosystems and often form symbiotic relationships with invertebrate fauna, including the alvinocarid shrimp, *Rimicaris exoculata* [4].

*R*. *exoculata* is highly abundant and widely distributed at black smoker chimneys along the Mid-Atlantic Ridge (MAR) hydrothermal vent fields; they live at temperatures between 10 and 20°C and reach aggregate densities up to 2,500 individuals m^-2^ [3, 5–8]. *R*. *exoculata* harbors two distinct chemoautotrophic ectosymbiotic bacterial communities. One community is located in the inner surfaces of its gill chamber and mainly composed of *Epsilon*, *Gamma*, *Zeta* and *Deltaproteobacteria*, known as sulfide, iron or methane oxidizers that obtain nutrition from the vent activity [8–16]. *R*. *exoculata* undergoes molting phases in which all of the gill epibionts are lost at each molt over the course of 10 days [17]. Most of the gill chamber symbionts appear to be locally acquired, likely being obtained from the surrounding seawater [18, 19]. Previous biogeographic analyses tend towards a local distribution of the symbionts, suggesting possible horizontal transmission [14].

A second symbiotic community was characterized in the digestive system that includes the gut and stomach [20, 21]. This community is primarily composed of bacteria from classes *Mollicutes*, *Deferribacteres*, *Epsilon* and *Gammaproteobacteria*, including both resident and externally derived bacteria that are likely introduced by *R*. *exoculata*’s grazing on various particles found in the surrounding environment [20]. Similar to the gill chamber, the stomach is subjected to molting; however, the gut is not, and is nearly emptied after each molting phase [17].

Recent genetic studies of the *R*. *exoculata* host sought to infer spatial and temporal population structure across several vents along the MAR [22, 23]. Using both mitochondrial and microsatellite markers, Teixeira and others found a lack of structure across the ranges investigated, suggesting high dispersal capacities and recent population expansion of *R*. *exoculata*. These findings identify *R*. *exoculata* as either one of the first known hydrothermal vent taxa that shows no barriers to dispersal across such a large geographic extent (more than 7,000km), or as a taxon with restrictions to gene flow too recent to have left a signature of drift on possibly very large populations [23]. Previous characterizations of the bacterial communities associated to the gill chamber were based on 16S ribosomal rRNA gene (16S) and identified a similar lack of geographic separation for *Gamaproteobacteria*, whereas geographic clustering was detected for *Epsilonproteobacteria* across four vents along the MAR [14]. Similarly, communities associated to the gut were shown to contain specific lineages of *Mollicutes* and *Deferribacteres* that remained undetected in the surrounding water [18], yet exhibited differentiation among sites [24]. The geographic clustering of specific bacterial lineages within a seemingly panmictic host suggests either horizontal transmission (environmental selection) of prokaryote communities as a result of unique vent geochemistry among locations [14], or vertical transmission (inheritance), in which prokaryote communities exhibit genetic differentiation not yet evident in the host [24].

Similar to the scaled specialization hypothesis (SSH), which postulates that the short generation time of taxocenes with smaller individuals may lead to a more rapid and thorough differentiation along environmental gradients [25], it has been hypothesized that the shorter generation time and/or smaller effective population size of some symbionts, particularly when engaged in a very tight association to their host, would lead to a more rapid establishment of genetic differentiation than for their hosts [26]. As some lineages within the prokaryotic communities that accompany *R*. *exoculata* seemingly show geographic clustering, this is an interesting hypothesis to test on this biological system.

In the present study, we used DNA metabarcoding surveys of prokaryotic communities within the digestive organs of *R*. *exoculata* to uncover signatures of genetic differentiation and population structure not seen in the host. We performed 454 sequencing of the 16S rRNA gene to compare bacterial assemblages associated with two organs (digestive tract and stomach), three molt colors that corresponding to molt phase of adult shrimp (white, red, black), and three life stages (eggs, juveniles, adults). Samples were retrieved from *R*. *exoculata* collected from three geochemically distinct vent locations, Rainbow, Trans-Atlantic Geotraverse (TAG) and Logatchev, all of which are located along the Mid-Atlantic Ridge.

Our strategy aimed to investigate significant differences occurring at the overall digestive community level before scaling down to operational taxonomic units (OTUs, identification of a taxonomic group when only DNA sequences are available, [27]). The apparent color of the adult carapace is a result of the accumulation of mineral deposits and typically ranges from white to red, grey and black; colors correspond with the settlement, growth and proliferation of bacterial communities [17]. Dark red and black are the colors typically seen before molting, after which the carapace returns to white for the start of the next cycle. We also investigated whether communities would differ among life stages, as digestive tract communities are not subjected to the molting cycle and most likely encompass persistent lineages. Previous examinations of the specific organs (gut, [20, 21]) and life stages (eggs vs. juveniles vs. adults for the gill chamber only, [19]) based on clone libraries identified the presence of specific bacterial lineages that may alternate across life stages. Therefore, we explored whether overall bacterial communities follow similar patterns seen in these previous studies.

Through specific analyses of OTUs, we determined whether OTUs 1) segregate among vents, possibly because of environmental selection (a scenario favoring horizontal transmission), or alternatively 2) segregate among host populations, possibly because of previously undetected restriction to gene flow (a scenario favoring vertical transmission).

## Materials and methods

### Animal collections and sampling processing for pyrosequencing

**Fig 1:**
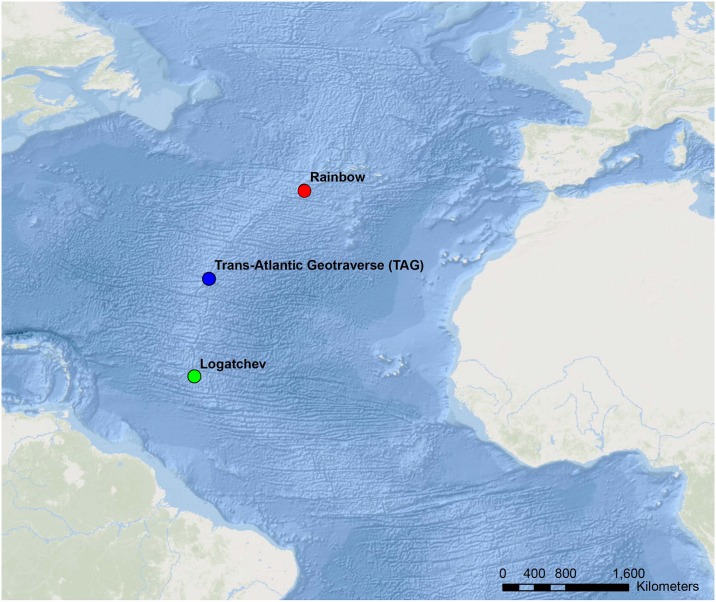
Geographic locations of three hydrothermal vents along the Mid-Atlantic Ridge (MAR). Hydrothermal vent locations from where *Rimicaris exoculata* were sampled for this study.

*Rimicaris exoculata* were collected via slurp gun from three hydrothermal vent field locations in 2005 (EXOMAR), 2007 (SERPENTINE and MoMARDREAM) and 2008 (MoMAR08) with the aid of remotely operated vehicle *Victor 6000* and human occupied vehicle *Nautile* aboard research vessels *Pourquoi pas*? and *L’Atalante* (S1 Table, Fig 1). No specific permissions were required to perform collections at any of these locations because the work performed here did not involve endangered or protected species. Once collected, adult individuals were brought aboard and dissected to remove the digestive tract and stomach, all done under sterile conditions. These collections resulted in an initial total of 50 samples. All samples were preserved at -80°C and transported back to the laboratory (see [23] for complete sampling protocol). Juvenile *R*. *exoculata*, measuring about 1.5 cm, were collected at the periphery of adult aggregations using the slurp gun. Once brought to the surface, juveniles were identified via dissecting scope and preserved at -80°C for dissection on shore using microscopes and micro-dissection tools to separate the stomach and the gut. All dissections occurred under sterile conditions. Females with eggs were obtained only from the Logatchev vent, which resulted in three egg samples each encompassing a small grape of eggs collected from three different mothers for analysis. DNA extractions of bacterial communities were performed using the Fast DNA-SPIN kit for soil, following the protocol provided by the manufacturer (MP Biomedicals, France).

Analysis of prokaryotic diversity and community composition was performed using 454 pyrosequencing of a 450-bp fragment of the 16S ribosomal rRNA gene (16S). Amplification of the V3-V4 region was performed using barcoded fusion primers (V3 forward: 16Sar: 5′-ACTCCTACGGGAGGCAG-3′, V4 reverse 16Sbr: 5′-TACNVRRGTHTCTAATYC-3′; [28]) with Roche-454 A Titanium sequencing adapters and unique eight-base barcode sequences to differentiate each sample pooled for sequencing. Fragments were amplified from each sample with Platinum High Fidelity Taq (Invitrogen, CA, USA) using 50ng of genomic DNA template. PCR conditions involved a 5 minute denaturation at 94°C followed by 35 cycles of 30 seconds at 94°C, 45 seconds at 44°C, 1 minute at 72°C and a final extension of 10 minute at 72°C. Positive and negative controls (ultrapure water only) were included for all amplification reactions, and tested as expected. The amplified products were quantified by fluorimetry with “PicoGreen” kit (Invitrogen, CA, USA), pooled at equimolar concentrations and sequenced in the A direction with GS 454 FLX Titanium chemistry, per manufacturer’s instructions (Roche, 454 Life Sciences, Brandford, CT, USA).

### Bioinformatics data processing and analyses

The resulting 16S pyrosequenced reads were processed, clustered and taxonomically assigned using the Quantitative Insights into Microbial Ecology (QIIME) v. 1.7.0 pipeline [29]. A workflow of all commands is available in the supporting materials (S1 File). After processing and quality filtering, 31 of the 50 samples originally collected had sequence counts of ≥500; therefore, the dataset composed of those 31 samples was used for downstream analyses (S2 and S3 Tables). Dataset demultiplexing and quality checks were performed using the split_libraries.py script to remove low quality and short sequences (< 250bp). Next, reads were clustered into *de novo* operational taxonomic units (OTUs) using UCLUST [30] via the pick_otus.py, with a pairwise sequence identity cut off value of 97%. Representative sequences for the clusters were then generated using the pick_rep_set.py script. Additionally, chimeras and “quasi-singletons” (sequences appear < 3 times in the dataset) were removed using USEARCH v.6.1 [30]. Finally, taxonomy was assigned to the representative sequences using the RDP classifier method implemented through the assign_taxonomy.py script in QIIME, with the Greengenes 2013 reference database [31] at 80% threshold identity [32].

Multiple rarefactions were applied to obtain standardized estimates of alpha diversity using Chao1 measurement for species richness [33]. Chao1 was calculated using the *specpool* function in the Vegan community ecology package (v.2.3–2) executed in the software R (v.3.2.2). Analysis of Similarities (ANOSIM) and Permutational Analysis of Variance (PERMANOVA) were performed, to test for the null hypothesis of homogeneity of bacterial communities among sample groups (i.e. organs, molts, life stages) using PAST (Paleontological statistics software package, [34]). ANOVA testing of OTU frequency among groups was performed using the group_significance.py script in QIIME. Finally, Multidimensional Scaling (MDS) analyses were performed based on Bray-Curtis distances in PAST.

Either as a consequence of amplification failures during the library preparation stage or low DNA concentration in some of the samples, the initial balanced sampling design resulted in uneven sample numbers for some “treatment” categories (i.e. organs, molts and life stages) after the quality filtering process of the data. This impeded the nested design initially planned for ANOSIM and PERMANOVA statistical testing, which was thus performed on treatments that had equal or near equal representation of each category. The strategy for testing the homogeneity of communities is shown in S4 Table and was performed 1) among vents using digestive tract samples 2) between digestive tracts and stomachs using samples from both TAG and Logatchev, 3) among eggs, juveniles and adults using samples from Logatchev and 4) among white, red and black molts using digestive tracts samples collected from Rainbow.

As statistical testing for differentiation of communities by vent was performed using only sequences from the digestive tract, clustering networks were generated using these same sequences that matched to *R*. *exoculata* ectosymbionts. The purpose of the networks was not to detail the evolutionary history of lineages, which would require a longer fragment size for robustness, but to allow visualization of the geographic clustering patterns of bacterial lineages across the three vent sites from which *R*. *exoculata* was collected (*see* [35]). Sequences that were ascribed to the ectosymbiont OTUs were filtered from the main dataset using the filter_fasta.py command in QIIME. Alignment and processing of the sequences sets was completed executing the make_alignment.py and filter_alignment.py commands via the PYNAST method [36] at the default parameters. Alignments were performed with the aid of the SILVA 123 core aligned reference dataset ([37], http://www.arb-silva.de/). After filtering, alignments were imported into DNAsp v.5.10.1 [38] where identical sequences were clustered into haplotypes. Haplotype outputs were exported in Roehl format for network calculation and drawing in Network by Fluxus (http://www.fluxus-engineering.com). Due to the presence of multiple possible characters for numerous sites, we implemented the Median-joining procedure [39].

## Results

Our pyrosequencing of the bacterial communities associated to the vent shrimp *Rimicaris exoculata* produced a total of 366,134 raw sequence reads. The raw reads were quality filtered, resulting in 106,179 sequences that had a mean sequence length of 406-bp. Next, the sequence dataset was clustered into 1,726 representative OTUs; 1,621 (94%) of these OTUs were taxonomically assigned to homologs in the RDP database. Those OTUs that remained unassigned (6.1%) were discarded for remaining analyses.

### OTUs assigned only to the domain level

Of the assigned OTUs, 1,063 (about 65%) were described only to the domain level, referred to as ‘bacteria’, and had an average length of 320-bp. Further inspection identified that seven of the 31 samples had >25% of the sequences assigned to ‘bacteria’ (S5 Table). As these samples represented various vents, organs and molts, there was no specific sample type to which the unclassified bacteria were most common.

Given their large percentage in the total dataset, 100 of the most common ‘bacteria’ OTUs (those to which the highest number of sequences were assigned) were individually blasted against NCBI’s global public database. Only two of the 100 received hits to isolates of known *R*. *exoculata* symbionts, revealing the limited number of known lineages in the pool assigned only to the domain. In attempt to identify possible phylogenetic clustering of the unclassified bacteria, the same 100 OTUs were aligned with *R*. *exoculata* ectosymbiont sequences from classes *Epsilonproteobacteria*, *Gammaproteobacteria*, *Mollicutes* and *Deferribacteres*, which were identified by previous researchers [14, 20, 24]. The overall findings showed no specific clustering with any of the known symbionts (S1–S4 Figs), which confirms that these sequences are too ambiguous to be reliably assigned to known taxonomy. The large percentage of the unclassified bacteria prompted us to perform statistical testing with and without this group. While there were differences in species richness estimates between the two datasets, the results of the statistical testing were congruent between the datasets including and excluding the unclassified bacteria. As the unclassified bacterial may include undetected chimeras, artifacts produced from 454-sequencing, or partial archaeal sequences or host DNA, we chose to focus on the conservative dataset that excluded these unclassified bacteria, which was composed of 557 OTUs assigned beyond the domain level. However, statistical analyses that include unclassified bacteria are detailed in the supporting materials (S6–S8 Tables, S5 and S6 Figs) and show similar results obtained with the dataset that excludes the unclassified bacteria.

### Comparisons among vents, organs, life stages and molts

**Fig 2:**
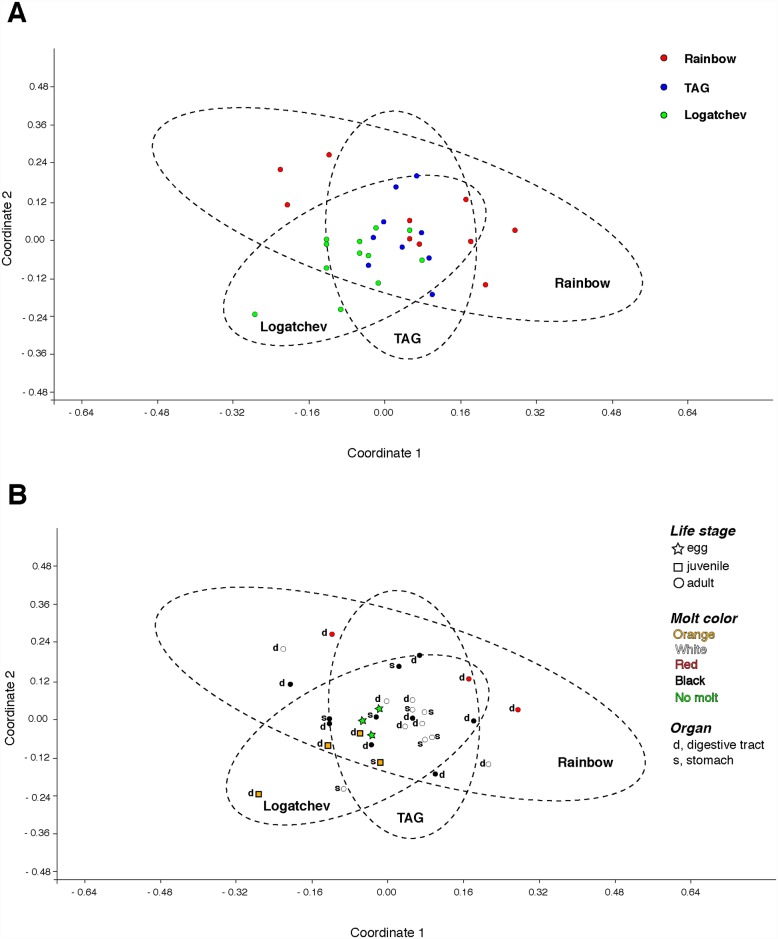
Multidimensional scaling (MDS) analyses for *Rimicaris exoculata* bacterial communities. (A) Clustering pattern by vents, with colored circles representing the vent location from where each sample was collected. (B) Clustering pattern by vent, with colored shapes and letters denoting the specific categories for each sample. Each MDS was implemented using Bray-Curtis similarity matrices, calculated in PAST [34]. The 95% concentration ellipses estimate a region where 95% of the population points are expected to fall. “Orange” indicates juveniles, whose carapace is an orange color.

Species richness (Chao1) values for each category by vent are shown in Table 1. Across vents, species richness was highest at TAG and lowest for Rainbow. Testing across vents supported a spatial differentiation of bacterial communities (p *<* 0.005, Table 2), with Rainbow as the most dissimilar (pairwise p ≤0.002, S9 Table) and most geographically distant from the other vents (Fig 1). ANOSIM testing additionally revealed an R value of 0.630 (S9 Table), which supports a separation of communities, despite the existence of overlap [40]. Further, ANOVA testing identified that OTUs matching the class *Deferribacteres* showed significant heterogeneity across vents and were more abundant at Rainbow (S2 File). The spatial organization of samples per vent, organ, life stage and molt color is shown in Fig 2. While the data identifies three clusters with overlapping communities, statistical testing supported a differentiation of bacterial communities across vents.

**Table 1:** Alpha diversity indices, species count and species richness (Chao1 and standard error).

Vent	Group		Number of Samples	Species Count	Chao1 ± SE
**Rainbow (n = 10)**	Organ	Stomach	0	n/a	n/a
		Digestive Tract	10	228	455.82 ± 14.95
	Molt	White	4	154	282.65 ± 35.05
		Red	3	118	269.74 ± 48.52
		Black	3	130	253.13 ± 36.40
	Life Stage	Eggs	0	n/a	n/a
		Juvenile	0	n/a	n/a
		Adult	10	228	455.82 ± 14.95
**TAG (n = 9)**	Organ	Stomach	4	226	571.54 ± 80.96
		Digestive Tract	5	330	900.32 ± 109.30
	Molt	White	4	155	280.28 ± 34.62
		Red	0	n/a	n/a
		Black	5	327	540.59 ± 39.54
	Life Stage	Eggs	0	n/a	n/a
		Juvenile	0	n/a	n/a
		Adult	9	375	574.55 ± 36.52
**Logatchev (n = 12)**	Organ	Stomach	5	261	363.56 ± 22.82
		Digestive Tract	4	257	339.11 ± 19.12
	Molt	White	3	149	447.68 ± 89.80
		Red	0	n/a	n/a
		Black	2	203	431.44 ± 55.50
	Life Stage	Eggs	3	192	256.22 ± 17.38
		Juvenile	4	272	370.10 ± 22.31
		Adult	5	262	444.21 ± 37.75

**Table 2:** Testing for homogeneity of bacterial communities. Testing was performed using Bray-Curtis Similarity Index of Analysis of similarities (ANOSIM) and Permutational Analysis of Variance (PERMANOVA). Significance was detected at p < 0.05 for vents and life stages.

All vents	ANOSIM	PERMANOVA
*Ho*: *no difference between vents*	*p < 0*.005*	*p < 0*.005*
**Logatchev**		
*Ho*: *no difference between digestive tract and stomach*	*p = 0*.*066*	*p = 0*.*117*
*Ho*: *no difference between various life stages*	*p = 0*.*011*‡	*p < 0*.*005*‡
**TAG**		
*Ho*: *no difference between digestive tract and stomach*	*p = 0*.*974*	*p = 0*.*562*
**Rainbow**		
*Ho*: *no difference between white*, *red and black molts*	*p = 0*.*606*	*p = 0*.*684*

* Rainbow is different from other vents

^‡^ eggs are different from other life stages

Comparisons across life stages at Logatchev revealed that species richness was highest for adults, followed by juvenile guts (Table 1, S7 Fig). Support for community separation among life stages was found (p < 0.012, Table 2), with eggs having the most dissimilar community compared to adults or juveniles (S9 Table). Despite these findings, there was evidence for an overlap of communities between life stages (R value = 0.373, S9 Table). Additionally, *Gammaproteobacteria* was found to be significantly more abundant in eggs (Supporting excel 1); it should be noted that *Gammaproteobacteria* are commonly found from eggs, as opposed to *Mollicutes* and *Deferribacteres*, which are rare or absent from eggs.

Finally, there was no significant differentiation of bacterial communities characterized among organs (digestive tracts and stomachs), at either Logatchev or TAG, nor across the three molts stages sampled in Rainbow (p = 0.606 Table 2, S9 Table).

### Most commonly assigned OTU classes

**Fig 3:**
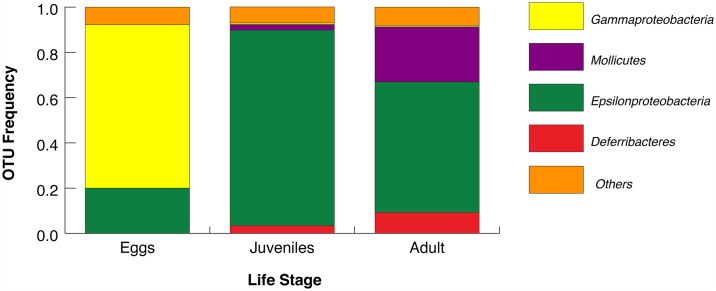
Frequencies of identified bacterial classes across the three life stages at Logatchev. Four main classes are identified across *Rimicaris exoculata* life stages. “Other classes” contain nine or more less common groups.

The total frequency of OTUs assigned to various bacterial classes by life stage at Logatchev is shown in Fig 3 and detailed in S10 Table. These classes have been previously identified as ectosymbionts of *R*. *exoculata*: *Deferribacteres*, *Mollicutes*, *Epsilon* and *Gammaproteobacteria* [19, 20, 24]. *Gammaproteobacteria* were dominant in eggs (Fig 3), while *Epsilonproteobacteria* were dominant in juvenile and adult guts, a result that differs slightly from previous analyses [20]. Regarding adults from Rainbow for which there were only digestive tracts available, 85.8% of OTUs were assigned to *Deferribacteres*, while adult communities from TAG were dominated by *Epsilonproteobacteria* (40.3%) and *Mollicutes* (24.5%) (S10 Table).

The largest OTUs (those OTUs defined as having > 500 sequences ascribed) were taxonomically assigned to three *R*. *exoculata* ectosymbiont classes listed in Table 3 and described in detail below.

**Table 3:** Qualitative results for digestive tract sequences assigned to the most common operation taxonomic units (OTUs). Common OTUs are defined as OTUs with >500 sequences assigned. The table illustrates the OTU name, assigned taxonomic identities, number of sequences, and the relative abundance of each OTU at each vent, as a percentage.

OTU	Identified Phyla	Identified Class	Sequence Count	Rainbow %	TAG %	Logatchev %
denovo 10553	*Deferribacteres*	*Deferribacteres*	504	1.14	1.18	0.07
denovo 6700	*Deferribacteres*	*Deferribacteres*	19,734	83.47	11.47	9.35
denovo 6152	*Tenericutes*	*Mollicutes*	691	0.00	2.75	0.39
denovo 2977	*Tenericutes*	*Mollicutes*	3,480	5.25	10.18	0.66
denovo 10123	*Proteobacteria*	*Epsilonproteobacteria*	668	0.05	1.53	2.82
denovo 5619	*Proteobacteria*	*Epsilonproteobacteria*	1,175	0.65	2.93	3.39
denovo 2874	*Proteobacteria*	*Epsilonproteobacteria*	863	1.19	2.45	0.52
denovo 529	*Proteobacteria*	*Epsilonproteobacteria*	1,690	0.80	1.69	10.77
denovo 10556	*Proteobacteria*	*Epsilonproteobacteria*	6,543	0.68	24.14	6.82
denovo 8992	*Proteobacteria*	*Epsilonproteobacteria*	4,976	0.75	5.23	34.08
denovo 7520	*Firmicutes*	*Clostridia*	3,741	0.00	15.87	0.00
Total	---	---	---	93.98	79.43	68.88

#### Deferribacteres

**Fig 4:**
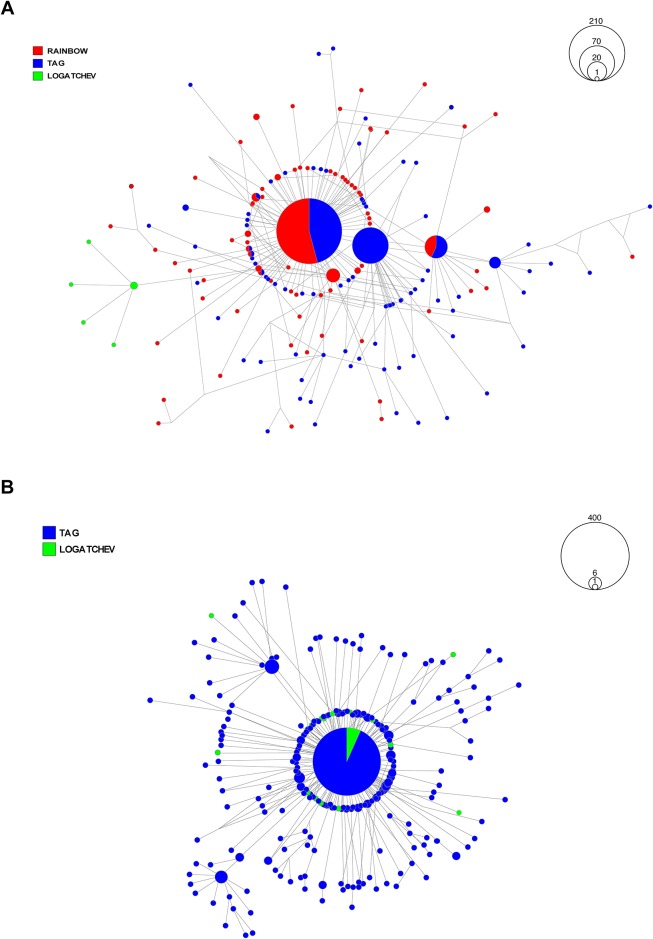
Geographic networks for operational taxonomic units (OTUs) assigned to bacterial classes *Deferribacteres* and *Mollicutes*. (A) OTU “denovo 10553”, assigned to *Deferribacteres*, was composed of 504 sequences. (B) OTU “denovo 6152”, assigned to *Mollicutes*, was composed of 691 sequences. Networks were drawn with median-joining calculation to illustrate the clustering of haplotypes; links are not proportional to the number of mutations and therefore, do not illustrate evolutionary divergence between the nodes.

Two OTUs with > 500 sequences were assigned to class *Deferribacteres* (denovo 10553 and denovo 6700, Table 3). Due to the technical constraints of processing 19,984 sequences ascribed to denovo 6700 OTU, we instead generated a list of the most common haplotypes (haplotypes with ≥20 sequences) along with the percentages of sequences from each vent (S11 Table). For denovo 10553, we constructed a cluster network to illustrate the geographic distributional pattern of haplotypes across all three vents. For both OTUs, sequences from Rainbow and TAG emerged as highly dominant (Fig 4A, S11 Table). For denovo 6700, dominant haplotypes were well represented by sequences from Rainbow. For denovo 10553, both Rainbow and TAG shared the most prominent haplotype, though there was an abundance of haplotypes that were unique (unshared) to each vent. Logatchev was far less represented within the *Deferribacteres* OTUs; however, sequences from Logatchev formed a distinct and small group of haplotypes (Fig 4A).

#### Mollicutes

Two OTUs were assigned to the class *Mollicutes* (denovos 2977 and 6152, Table 3). For denovo 2977, the largest haplotypes were shared amongst all vents, with TAG representing 69.1% of all sequences (S11 Table). We constructed a network with sequences from denovo 6152 (Fig 4B), which identified Logatchev and TAG as sharing the most common haplotype. Despite the emergence of unique, unshared haplotypes from both vents, there was no clear pattern of separation by vent demonstrated by this class.

#### Epsilonproteobacteria

**Fig 5:**
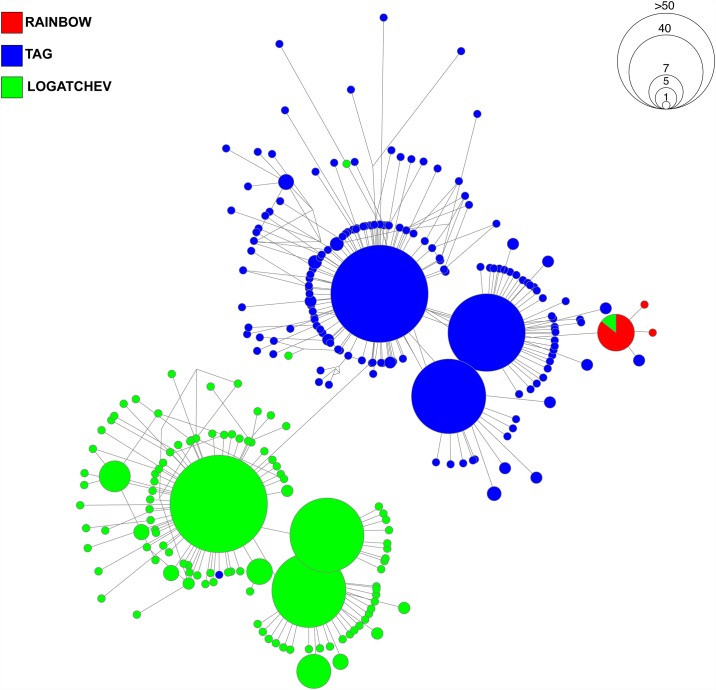
Geographic network for an operational taxonomic unit (OTU) assigned to bacterial class *Epsilonproteobacteria* (denovo 10123). OTU “denovo 10123” was composed of 668 sequences. Network was drawn with median-joining calculation to illustrate the clustering of haplotypes; links are not proportional to the number of mutations and therefore, do not illustrate evolutionary divergence between the nodes.

Six OTUs were assigned to the class *Epsilonproteobacteria* (Table 3). We produced a network from denovo 10123 and detailed the common haplotypes of the other OTUs in S11 Table. The largest haplotypes of the five other OTUs were dominated by sequences originating either from TAG, Logatchev or both (S11 Table). For denovo 10556, which was the largest *Epsilonproteobacteria* OTU in terms of the number of sequences, there were only four common haplotypes, but three of which were dominated by sequences from either TAG or Logatchev. This suggests that each vent has distinctive haplotypes for *Epsilonproteobacteria* that differ from the other vents. Further, the network for denovo 10123 identified a clear separation of distinct star-like clusters dominated either by Logatchev or TAG, also supporting segregation by vent (Fig 5). Rainbow was represented by <1% of all sequences belonging to three haplotypes.

#### Other OTUs

One common OTU was assigned to *Clostridia*, a bacterial class within the phylum *Firmicutes* that was only identified from TAG (Table 3).

We note an additional OTU, denovo 6909, that was assigned to the class *Gammaproteobacteria*. The number of sequences ascribed to denovo 6909 fell below 500 (n = 298). The low sequence number of this OTU prevents robust statistical inferences; however, as *Gammaproteobacteria* is a prominent class of bacteria within the symbiotic community of *R*. *exoculata*, we preferred to mention its presence. The constructed network illustrating that the most common haplotypes were almost entirely composed of sequences originating either from TAG or Logatchev (S8 Fig). Of the three major clusters of haplotypes, two were heavily dominated by sequences from TAG, while the other as dominated by sequences from Logatchev, supporting the separation by vent seen with *Epsilonproteobacteria*.

## Discussion

In this study, we focus on bacterial symbionts associated to the hydrothermal shrimp *Rimicaris exoculata*, rather than archaea ingested from the surrounding environment. Gut archaea have been found in environmental samples around hydrothermal vent chimneys and are ingested by the host; despite this, archaea were hypothesized to be expelled with the alimentary bolus [20]. Moreover, archaea are in low concentration in the gut (Durand, personal communication), limiting our ability to detect this low DNA concentration with 454 sequencing.

Compared with previous studies based on clone libraries, the deep sequencing of bacterial assemblages from organs, molt stages and life cycles of the *R*. *exoculata* digestive system provides both quantitative and qualitative insights on the diversity of prokaryote communities associated to this host. Our statistical testing did not support the differentiation of communities associated to distinct organs or molt stages, but did provide evidence for separation by vents and life stages (with Rainbow -digestive tracts only- and eggs exhibiting the most dissimilar communities). Through investigations focusing on both the community and OTU level, these results provide further insights into the transmission mode and time scale of connectivity of the studied bacterial epibionts that show a clear segregating pattern among vents, as well as the evolution of community composition throughout the life cycle.

### Bacterial communities over the life cycle

Our examinations of the bacterial composition within eggs, juveniles and adult stages from Logatchev identify that specific symbionts dominate, depending on the age of the host. The bacterial community present in eggs is dominated by *Gammaproteobacteria*, but also hosts *Espsilonproteobacteria* and very few *Mollicutes* (Fig 3, S10 Table). These results are in line with some findings reported by Guri *et al*. [19], with the exception of *Mollicutes*, which were not recovered. The present dataset shows *Epsilonproteobacteria* as the most common class in juveniles and adult guts, which was also reported by Durand *et al*. [20, 24], but without quantitative consideration. Additionally, adults exhibited higher species richness when compared to the other life stages at Logatchev (Table 1), which suggests an increased role of symbionts in adults, although further testing at other vents is needed to confirm this observation regarding the influence of life stage. Such accumulation does not seem to be strongly affected by the process of molting, as no significant differentiation was detected among molt stages (Table 2). This finding is unsurprising, as the digestive tract does not undergo molting despite a drastic reduction in symbiont load observed during molting.

### Physical and chemical composition of vents and the geographic differentiation of associated bacterial communities

Spatial analyses across vent locations revealed a significant heterogeneity among communities (Table 2), despite the overlap of bacterial community composition observed in the multidimensional analysis (Fig 2). This differentiation, most obvious at Rainbow, was also seen at the OTU level where some of the OTUs representing the classes *Deferribacteres* and *Epsilonproteobacteria* (Figs 4 and 5) also showed a clear geographical segregation to confirm the previous clone based analyses of Durand and others [24]. Vent locations along the Mid-Atlantic Ridge vary in the chemical composition of the rock basement, as well as the end member fluids that flow from the seafloor. Fluids from each vent contain iron, methane, hydrogen gas and sulfide; however, each location differs in the levels of each compound [41]. Rainbow is an ultra-mafic hosted site that contains high levels of iron, methane, hydrogen gas [41–43]. In contrast, TAG is a basalt-hosted site that is enriched in sulfide, but depleted in methane and hydrogen [44–47]. Logatchev represents an intermediate between ultra-mafic and basalt hosted sites, as it is enriched in methane, but less so in hydrogen and sulfides [48]. Ultra-mafic sites are present at slow spreading centers and are less frequently affected by seafloor eruptions and intrusions than basalt hosted sites; these events are known to cause large changes in fluid chemistry at vents [5, 49, 50]. Therefore, environmental processes and chemical compositions that include high levels of methane and hydrogen gas would establish Rainbow as *a priori* favorable for methanogens, methanotrophs and other hydrogen-oxidizing thermophiles [18]. Further, high sulfide concentrations present at basalt-hosted TAG would allow for the support of sulfide oxidizers [11, 41, 51]. Given this, one would expect that mineral particles and free living microorganisms ingested on ultramafic sites would differ from basaltic hosted ones. However, despite the location specific communities and OTUs observed here, shrimp from the basaltic site TAG were shown to support digestive bacterial communities that are intermediate between Rainbow and Logatchev (Fig 2). Thus, the composition of gut bacterial communities may be only partially driven by the chemistry of fluids released, but also depend on transmission and selection strategies of the epibionts whom may be sensitive to a broader diversity of environmental characteristics.

An example of an influential environmental variable of the ultramafic nature of sites is iron: *Deferribacteres* contains several iron reducing species that oxidize dissolved iron at vents [52–54] and as the dominant OTU class identified from digestive tracts obtained from Rainbow (Table 3, S10 Table), are likely to be driven by the much higher concentration of iron in the fluids at this vent. The heavy iron concentrations are also thought to be responsible for the red appearance of *R*. *exoculata* carapaces during the intermediate stages of the molt cycle [17, 55]. Additionally, iron oxides are present in the gut of *R*. *exoculata* at Rainbow [7] and while iron oxides are available in the environment, this *Deferribacteres* lineage has not yet been found the surrounding water. This suggests that the primary environment for these *Deferribacteres* is within the digestive tract of *R*. *exoculata*, an anatomical region not subjected to molting. Thus, this *Deferribacteres* lineage is likely member of a resident community that bears a high importance in the metabolism of the holobiont at Rainbow [20].

The geographic differentiation illustrated by *R*. *exoculata* bacterial communities could be attributed to two overarching and not mutually-exclusive causes. First, the distinct environmental conditions dominating each site may influence the abundance of bacterial strains available to be captured by and to survive in *R*. *exoculata*’s digestive system, supporting a scenario of horizontal acquisition of bacterial lineages. Second, isolated populations of bacteria not connected through migration may have diverged over several generations, leading to associated bacterial lineages being distinct beyond the 3% threshold used here to define OTU clusters. This isolation is compatible with both free-living lineages acquired horizontally and vertically, implying a barrier to gene flow directly impeding bacterial dispersal, or preventing the host migration.

### Differentiation of OTUs among sites and possible transmission scheme of symbionts

In agreement with previous investigations based on cloning, *Deferribacteres*, *Mollicutes*, *Epsilon* and *Gammaproteobacteria* emerge as the dominant classes in the digestive community, while other classes of *Proteobacteria* are less represented (Fig 3, S10 Table [20, 21, 24]). Bacterial aggregations of *Rimicaris exoculata* are likely composed of both horizontally and vertically transmitted bacteria and when compared to the multidimensional analyses at the community scale, networks detailing the arrangement of haplotypes provide a more specific focus on the geographic distribution of common bacterial OTUs.

The unique pattern of clear separation between Logatchev and TAG for *Epsilonproteobacteria* OTUs (Fig 5) is in line with observations reported by Petersen and others [14]. While *Epsilon* and *Gammaproteobacteria* are also present on eggs (Fig 3), favoring the hypothesis of a vertical transmission, each class is also found around *R*. *exoculata* aggregations in quantities that suggest that they are present as free-living forms, or from degrading tissues, feces or molts of congeners [14, 19]. This supports the possibility of horizontal transmission and differential filtration of the hosts depending on their local and most adapted metabolism. *Epsilon* and *Gammaproteobacteria* are also found on a diversity of hosts at vents, having led Petersen and others [14] to propose that free-living forms might disperse among vents to colonize unrelated hosts. The diversity characterized in the surrounding water does not include some of the digestive symbionts characterized here, including the prevalent *Mollicutes* and *Deferribacteres*, thereby supporting either vertical inheritance with spatial genetic differentiation, or the existence of a host recognition systems for promoting differential filtering of the strains present in the environment [13]. Such filtering mechanisms could lead to a similar composition of communities independent of the environmental conditions and for the purposes of fitting the basal metabolism of the holobiont. Further, differential distribution of symbionts across environmentally distinct vents may also occur through phenotypic plasticity of the host, which can differentially filter strains to optimize host metabolism under specific environmental conditions.

For those symbionts not yet found in the surrounding water, vertical transmission would be the most likely option; however, *Mollicutes* and *Deferribacteres* are almost not present in eggs, as shown here (S10 Table). Despite the current findings, the possibility of vertical transmission cannot be discarded, as evidence for this pathway may be present within a small number of OTUs undetected through past sequencing efforts, or alternatively, through transmission later in the egg or larval phases. Eggs may inherit vertically acquired bacteria at spawning, when the mother covers the eggs in a mucus layer that contains bacteria [56], or through trophallaxis with larvae or juveniles [24]. This mucus layer has been proposed to help fight pathogens while also attracting additional symbionts as a part of a host recognition process [19]. If vertically inherited, the separation by vent suggests that a signature of drift would be imprinted in the bacterial genome before that of the host.

## Conclusions

Using DNA metabarcoding of whole bacterial communities associated to *Rimicaris exoculata*, we provide evidence for the restriction to gene flow and geographic isolation of common symbionts, notably *Epsilonproteobacteria* and *Deferribacteres*. The community-wide differences uncovered here could be attributed to environmental factors such as distinctive site geochemistry combined with host filtering mechanisms, an inheritance aspect in which symbionts are vertically transmitted with limited migration among host populations, or a combination of both influences. Additional studies are required to tease apart which influence is strongest. The collection of surrounding water samples for further examination of free living bacteria might provide additional insights into the transmission mode of the lineages investigated in this study provided that i) the dominant seawater community would not prevent the detection, if existing, of free living strains that were also detected in the shrimp, and ii) the occurrence of strains expelled through feces or decomposition of aggregate animal remains would not impact the accurate description of free living communities. Further, more information on intermediate development stages (specifically larvae) is necessary to complete the picture of the evolution of bacterial communities through the life cycle, in addition to pressurized *in vivo* experiments on gravid females to collect mucus and test for the presence of vertically transmitted symbionts. Finally, investigation of the potential host-symbiont recognition capabilities would yield more insights into the community patterns that have been observed thus far.

## Supporting information

S1 Fig 16S Maximum Likelihood (ML) consensus boostrap tree for *Epsilonproteobacteria*. Tree illustrates 19 sequences obtained from Durand et al. 2015 [24] and 100 unclassifed bacteria OTUs (denovos), all from the hydrothermal vent shrimp *Rimicaris exoculata*. Bootstrap support (1000 replicates, Tamura-Nei model) is located either above or below the node. Sequences obtained from Durand et al. 2015, are shown in color (Rainbow = red, TAG = blue, Logatchev = green) and include the clone name and GenBank accession number. Denovo clusters containing multiple sequences were collapsed to reduce the length of the tree. These clusters are shown in bold and entitled “denovo”, followed by the number of sequences contained within the cluster, in parentheses. Scale is measured as number of substitutions per nucleotide site. The alignment was performed using Clustal W [57] in Geneious Pro v5.5.5 (Biomatters Ltd.) and the tree was calculated with the aid of MEGA 7 [58].
(PDF)

S2 Fig 16S Maximum Likelihood (ML) consensus boostrap tree for *Gammaproteobacteria*. Tree illustrates 16 sequences obtained from Petersen et al. 2010 [14] and 100 unclassifed bacteria OTUs (denovos), all from the hydrothermal vent shrimp *Rimicaris exoculat*a. Bootstrap support (1000 replicates, Tamura-Nei model) is located either above or below the node. Sequences obtained from Petersen et al. 2010, are shown in color (Rainbow = red, TAG = blue, Logatchev = green) and include the clone name and GenBank accession number. Denovo clusters containing multiple sequences were collapsed to reduce the length of the tree. These clusters are shown in bold and entitled “denovo”, followed by the number of sequences contained within the cluster, in parentheses. Scale is measured as number of substitutions per nucleotide site. The alignment was performed using Clustal W [57] in Geneious Pro v5.5.5 (Biomatters Ltd.) and the tree was calculated with the aid of MEGA 7 [58].
(PDF)

S3 Fig 16S Maximum Likelihood (ML) consensus boostrap tree for *Mollicutes*. Tree illustrates 13 sequences obtained from Durand et al. 2015 [24] and 100 unclassifed bacteria OTUs (denovos), all from the hydrothermal vent shrimp *Rimicaris exoculata*. Bootstrap support (1000 replicates, Tamura-Nei model) is located either above or below the node. Sequences obtained from Durand et al. 2015, are shown in color (Rainbow = red, TAG = blue, Logatchev = green) and include the clone name and GenBank accession number. Denovo clusters containing multiple sequences were collapsed to reduce the length of the tree. These clusters are shown in bold and entitled “denovo”, followed by the number of sequences contained within the cluster, in parentheses. Scale is measured as number of substitutions per nucleotide site. The alignment was performed using Clustal W [57] in Geneious Pro v5.5.5 (Biomatters Ltd.) and the tree was calculated with the aid of MEGA 7 [58].
(PDF)

S4 Fig 16S Maximum Likelihood (ML) consensus boostrap tree for *Deferribacteres*. Tree illustrates 18 sequences obtained from Durand et al. 2015 [24] and 100 unclassifed bacteria OTUs (denovos), all from the hydrothermal vent shrimp *Rimicaris exoculata*. Bootstrap support (1000 replicates, Tamura-Nei model) is located either above or below the node. Sequences obtained from Durand et al. 2015, are shown in color (Rainbow = red, TAG = blue, Logatchev = green) and include the clone name and GenBank accession number. Denovo clusters containing multiple sequences were collapsed to reduce the length of the tree. These clusters are shown in bold and entitled “denovo”, followed by the number of sequences contained within the cluster, in parentheses. Scale is measured as number of substitutions per nucleotide site. The alignment was performed using Clustal W [57] in Geneious Pro v5.5.5 (Biomatters Ltd.) and the tree was calculated with the aid of MEGA 7 [58].
(PDF)

S5 Fig Multidimensional Scaling (MDS) analyses for *Rimicaris exoculata* bacterial communities, including unidentified bacteria. (A) Clustering pattern by vents, with colored circles representing the vent location from where each sample was collected. (B) Clustering pattern by vent, with colored shapes and letters denoting the specific categories for each sample. Each MDS was implemented using Bray-Curtis similarity matrices, calculated in PAST [34]. The 95% concentration ellipses estimate a region where 95% of the population points are expected to fall. “Orange” indicates juveniles, whose carapace is an orange color. Note: the number of samples is 33, rather than 31, as the addition of unidentified bacteria allows us to include two additional samples that have > 500 sequences assigned.
(PDF)

S6 Fig Frequencies of identified bacterial classes for the three life stages at Logatchev, including unidentified bacteria. Three main classes are identified across *Rimicaris exoculata* life stages. “Other classes” contain nine or more less common groups.
(PDF)

S7 Fig Species count and species richness for molt colors at Rainbow (A) and life stages at Logatchev (B). (PDF)

S8 Fig Geographic network for Operational Taxonomic Unit (OTU) assigned to *Gammaproteobacteria*. denovo 6909 was composed of 298 sequences. Network was drawn using star-contraction before applying the median-joining calculation to illustrate the clustering of haplotypes; links are not proportional to the number of mutations and therefore, do not illustrate evolutionary divergence between the nodes.
(PDF)

S1 File QIIME workflows and commands. (DOCX)

S2 File ANOVA testing of OTU frequency among groups, performed using the group_significance.py script in QIIME [29]. (XLS)

S1 Table Geographic coordinates, depth, year(s) and number of samples sequenced for *Rimicaris exoculata* collections from three vents along the Mid-Atlantic Ridge (MAR) (DOCX)

S2 Table *Rimicaris exoculata* sample counts by organs, molts and life stage categories. *n* refers to the number of total samples collected from each vent. Only adults were identified by molt color.
(DOCX)

S3 Table Metadata for each sample. (DOCX)

S4 Table Strategy for statistically testing the rejection of the null hypothesis of homogeneity of bacterial communities across molt stages, organs, life stages and vents. (DOCX)

S5 Table Counts and percentages of OTUs that were described only to domain level ‘bacteria’ for each sample (unclassified bacteria). Seven samples had > 25% of the OTUs described as ‘Bacteria’; all seven samples were taken from adult *R*. *exoculata*.
(DOCX)

S6 Table ANOSIM test results for dataset including unclassified bacteria. The Bray-Curtis similarity index at 9999 permutations was used (*) defines significant relationships at p = 0.05.
(DOCX)

S7 Table Alpha diversity indices, species count and species richness (Chao1 and standard error) for dataset including unclassified bacteria. (DOCX)

S8 Table Frequency of OTUs assigned to class for each vent, including unclassified bacteria. *asterisk denotes less represented phyla that include classes of Acidobacteria, Actinobacteria, Bacteroidetes, Chlorobi, Cyanobacteria, Firmicutes, GB02, SR1 and other proteobacteria.
(DOCX)

S9 Table ANOSIM and PERMANOVA results excluding unclassified bacteria. The Bray-Curtis similarity index at 9999 permutations was used (*) defines significant relationships at p = 0.05.
(DOCX)

S10 Table Frequency of OTUs assigned to class for each vent, excluding unclassified bacteria. *asterisk denotes less represented phyla that include classes of Acidobacteria, Actinobacteria, Bacteroidetes, Chlorobi, Cyanobacteria, Firmicutes, GB02, SR1 and other proteobacteria.
(DOCX)

S11 Table Qualitative results of the most common haplotypes. Most common haplotypes are defined as those represented by ≥20 sequences within common operational taxonomic units (OTU) for which networks were not drawn. Each common haplotype is listed by name, number of sequences within each haplotype, and the percentage of sequences from each vent.
(DOCX)

## References

[1] Childress J, Fisher C. The biology of hydrothermal vent animals: physiology, biochemistry, and autotrophic symbioses. Oceanography and marine biology: an annual review. 1992;30:337–441.

[2] Tunnicliffe V, Fowler M. Influence of sea-floor spreading on the global hydrothermal vent fauna. Nature. 1996;379(6565):531–3.

[3] Desbruyères D, Biscoito M, Caprais J-C, Colaço A, Comtet T, Crassous P, et al Variations in deep-sea hydrothermal vent communities on the Mid-Atlantic Ridge near the Azores plateau. Deep-Sea Research I. 2001;(1325–1346).

[4] Williams A, Rona P. Two new caridean shrimps (Bresiliidae) from a hydrothermal field on the Mid-Atlantic Ridge. Journal of Crustacean Biology. 1986:446–62.

[5] Schmidt C, Le Bris N, Gaill F. Interactions of deep-sea invertebrates with their environment: the case of *Rimicaris exoculata*. Journal of Shellfish Research. 2008;27:79–90.

[6] Van Dover C, Fry B, Grassle J, Humphris S, Rona P. Feeding biology of the shrimp *Rimicaris exoculata* at hydrothermal vents on the Mid-Atlantic Ridge. Marine Biology. 1988;98:209–16.

[7] Zbinden M, Le Bris N, Gaill F, Compère P. Distribution of bacteria and associated minerals in the gill chamber of the vent shrimp *Rimicaris exoculata* and related biogeochemical processes. Marine Ecology Progress Series. 2004;284:237–51.

[8] Zbinden M, Shillito B, Le Bris N, de Montlaur C, Roussel E, Guyot F, et al New insights on the metabolic diversity among the epibiotic microbial community of the hydrothermal shrimp *Rimicaris exoculata*. Journal of Experimental Marine Biology and Ecology. 2008;359:131–40.

[9] Emerson D, Rentz J, Lilburn T, Davis R, Aldrich H, Chan C, et al A Novel Lineage of Proteobacteria Involved in Formation of Marine Fe-Oxidizing Microbial Mat Communities. PLoS One. 2007;2(8):e667. 17668050 10.1371/journal.pone.0000667 PMC1930151

[10] Emerson D, Moyer C. Neutrophilic Fe-oxidizing bacteria are abundant at the Loihi Seamount hydrothermal vents and play a major role in Fe oxide deposition. Applied and Environmental Microbiology. 2002;68:3085–93. 10.1128/AEM.68.6.3085-3093.2002 12039770 PMC123976

[11] Hügler M, Petersen J, Dubilier N, Imhoff J, Sievert S. Pathways of carbon and energy metabolism of the epibiotic community associated with the deep-sea hydrothermal vent shrimp *Rimicaris exoculata*. PLoS One. 2011;6(1):e16018 10.1371/journal.pone.0016018 21249205 PMC3017555

[12] Inagaki F, Takai K, Nealson K, Horikoshi K. *Sulfurovum lithotrophicum* gen. nov., sp. nov., a novel sulfur-oxidizing chemolithoautotroph within the e-Proteobacteria isolated from Okinawa Trough hydrothermal sediments. International Journal of Systematic and Evolutionary Microbiology. 2004;54(5):1477–82. 15388698 10.1099/ijs.0.03042-0

[13] Jan C, Petersen J, Werner J, Teeling H, Huang S, Glöckner F, et al The gill chamber epibiosis of deep-sea *Rimicaris exoculata*: an in-depth metagenomic investigation and discovery of Zetaproteobacteria. Environmental microbiology. 2014;16(9). 10.1111/1462-2920.12406 24447589

[14] Petersen J, Ramette A, Lott C, Cambon-Bonavita M, Zbinden M, Dubilier N. Dual symbiosis of the vent shrimp *Rimicaris exoculata* with filamentous gamma- and epsilonproteobacteria at four Mid-Atlantic Ridge hydrothermal vent fields. Environmental microbiology. 2010;12(8):2204–18. 10.1111/j.1462-2920.2009.02129.x 21966914

[15] Polz M, Cavanaugh C. Dominance of one bacterial phylotype at a Mid-Atlantic Ridge hydrothermal vent site. Proceedings in the National Academy of Science USA. 1995;92:7232–6. 10.1073/pnas.92.16.7232 PMC41313 7543678

[16] Ponsard J, Cambon-Bonavita M, Zbinden M, Lepoint G, Joassin A, Corbari L, et al Inorganic carbon fixation by chemosynthetic ectosymbionts and nutritional transfers to the hydrothermal vent host-shrimp *Rimicaris exoculata*. The ISME Journal. 2013;7(1):96–109. 10.1038/ismej.2012.87 22914596 PMC3526180

[17] Corbari L, Zbinden M, Cambon-Bonavita M, Gaill F, Compère P. Bacterial symbionts and mineral deposits in the brachial chamber of the hydrothermal vent shrimp *Rimicaris exoculata*: relationship to moult cycle. Aquatic Biology. 2008;1:225–38.

[18] Flores G, Campbell J, Kirshtein J, Meneghin J, Podar M, Steinberg J, et al Microbial community structure of hydrothermal deposits from geochemically different vent fields along the Mid-Atlantic Ridge. Environmental microbiology. 2011;13(8). 10.1111/j.1462-2920.2011.02463.x 21418499

[19] Guri M, Durand L, Cueff-Gauchard V, Zbinden M, Crassous P, Shillito B, et al Acquisition of epibiotic bacteria along the life cycle of the hydrothermal shrimp *Rimicaris exoculata*. The ISME Journal. 2012;6:597–609. 10.1038/ismej.2011.133 21993397 PMC3280129

[20] Durand L, Zbinden M, Cueff-Gauchard V, Duperron S, Roussel E, Shillito B, et al Microbial diversity associated with the hydrothermal shrimp *Rimicaris exoculata* gut and occurance of a resident microbial community. FEMS Microbiology Ecology. 2010;71:291–303. 10.1111/j.1574-6941.2009.00806.x 19951370

[21] Zbinden M, Cambon-Bonavita M. Occurance of *Deferribacterales* and *Entomoplasmatales* in the deep-sea Alvinocarid shrimp *Rimicaris exoculata* gut. FEMS Microbiology Ecology. 2003;46:23–30. 10.1016/S0168-6496(03)00176-4 19719579

[22] Teixeira S, Cambon-Bonavita M, Serrão E, Desbruyéres D, Arnaud-Haond S. Recent population expansion and connectivity in the hydrothermal shrimp *Rimicaris exoculata* along the Mid-Atlantic Ridge. Journal of Biogeography. 2011;38:564–74.

[23] Teixeira S, Serrão E, Arnaud-Haond S. Panmixia in a fragmented and unstable environment: the hydrothermal shrimp Rimicaris *exoculata* disperses extensively along the Mid-Atlantic Ridge. PLoS One. 2012;7(6):e38521 10.1371/journal.pone.0038521 22679511 PMC3367947

[24] Durand L, Roumagnac M, Cueff-Gauchard V, Jan C, Guri M, Tessier C, et al Biogeographical distribution of *Rimicaris exoculata* resident gut epibiont communities along the Mid-Atlantic Ridge hydrothermal vent sites. FEMS Microbiology Ecology. 2015;91(10):1–15. 10.1093/femsec/fiv101 26324855

[25] Kaspari M, Stevenson B, Shik J, Kerekes J. Scaling community structure: how bacteria, fungi, and ant taxocenes differentiate along a tropical forest floor. Ecology. 2010;91(8):2221–6. 20836443 10.1890/09-2089.1

[26] Nieberding C, Olivieri I. Parasites: proxies for host genealogy and ecology? Trends in Ecology & Evolution. 2007;22(3):156–65. 17157954 10.1016/j.tree.2006.11.012

[27] Blaxter M, Mann J, Chapman T, Thomas F, Whitton C, Floyd R, et al Defining operational taxonomic units using DNA barcode data. 2005;360(1462):1935–43. 10.1098/rstb.2005.1725 PMC1609233 16214751

[28] Wang T, Qian P. Conservative fragments in bacterial 16S rRNA genes and primer design for 16S ribosomal DNA amplicons in metagenomic studies. PLoS One. 2009;4:e7401 10.1371/journal.pone.0007401 19816594 PMC2754607

[29] Caporaso J, Kuczynski J, Stombaugh J, Bittinger K, Bushman F, Costello E, et al QIIME allows analysis of high-throughput community sequencing data. Nature Methods. 2010;7(5):335–6. 10.1038/nmeth.f.303 20383131 PMC3156573

[30] Edgar R. Search and clustering orders of magnitude faster than BLAST. Bioinformatics. 2010;26(19):2460–1. 10.1093/bioinformatics/btq461 20709691

[31] DeSantis T, Hugenholtz P, Larsen N, Rojas M, Brodie E, Keller K, et al Greengenes, a chimera-checked 16S rRNA gene database and workbench compatible with ARB. Applied and environmental microbiology. 2006;72(7):5069–72. 10.1128/AEM.03006-05 16820507 PMC1489311

[32] Loudon A, Woodhams D, Wegener Parfrey L, Archer H, Knight R, McKenzie V, et al Microbial community dynamics and effect of environmental microbial reservoirs on red-backed salamanders (*Plethodon cinereus*). The ISME Journal. 2014;8:830–40. 10.1038/ismej.2013.200 24335825 PMC3960541

[33] Chao A. Nonparametric estimation of the number of classes in a population. Scandinavian Journal of Statistics. 1984;11(4):265–70.

[34] Hammer Ø, Harper D, Ryan P. PAST: Paleontological Statistics Software Package for Education and Data Analysis Palaeontol. Electronica. 2013;4:1–9.

[35] Aires T, Serrão E, Kendrick G, Durante C, Arnaud-Haond S. Invasion is a community affair: clandestine followers in the bacterial community associated to green algae, *Caulerpa racemosa*, track the invasion source. PLoS One. 2013;8(7):e68429 10.1371/journal.pone.0068429 23874625 PMC3713043

[36] Caporaso J, Bittinger K, Bushman F, DeSantis T, Andersen G, Knight R. PyNAST: a flexible tool for aligning sequences to a template alignment. Bioinformatics. 2010;26(2):266–7. 10.1093/bioinformatics/btp636 19914921 PMC2804299

[37] Pruesse E, Quast C, Knittel K, Fuchs B, Ludwig W, Peplies J, et al SILVA: a comprehensive online resource for quality checked and aligned ribosomal RNA sequence data compatible with ARB. Nucleic acids research. 2007;35(21):7188–96. 10.1093/nar/gkm864 17947321 PMC2175337

[38] Rozas J, Rozas R. DnaSP, DNA sequence polymorphism: an interactive program for estimating population genetics parameters from DNA sequence data. Computer applications in biosciences. 1995;11:621–5. 10.1093/bioinformatics/11.6.621 8808578

[39] Bandelt H-J, Forster P, Röhl A. Median-joining networks for inferring intraspecific phylogenies. Molecular biology and evolution. 1999;(16):1. 10.1093/oxfordjournals.molbev.a026036 10331250

[40] Ramette A. Multivariate analyses in microbial ecology. FEMS Microbiology Ecology. 2007;62:142–60. 10.1111/j.1574-6941.2007.00375.x 17892477 PMC2121141

[41] Charlou J, Donval J, Fouquet Y, Jean-Baptiste P, Holm N. Geochemistry of high H2 qnd CH4 vent fluids issuing from ultramafic rocks at the Rainbow hydrothermal field (36° 14'N, MAR). Chemical Ecology. 2002;191:345–59.

[42] Douville E, Charlou J, Oelkers E, Bienvenu P, Colon C, Donval J, et al The rainbow vent fluids (36°14’N, MAR): the influence of ultramafic rocks and phase separation on trace metal content in Mid-Atlantic Ridge hydrothermal fluids. Chemical Geology. 2002;184:37–48.

[43] Severmann S, Johnson C, Beard B, German C, Edmonds H, Chiba H, et al The effect of plume processes on the Fe isotope composition of hydrothermally derived Fe in the deep ocean as inferred from the Rainbow vent site, Mid-Atlantic Ridge, 36° 14’ N. Earth and Planetary Science Letters. 2004;225(1):63–76.

[44] Buck K, Sohst B, Sedwick P. The organic complexation of dissolved iron along the US GEOTRACES (GA03) North Atlantic Section. Deep Sea Research Part II: Topical Studies in Oceanography. 2015;116:152–65.

[45] Fouquet Y, Cambon P, Etoubleau J, Charlou J, Ondréas H, Barriga F, et al Geodiversity of Hydrothermal Processes Along the Mid-Atlantic Ridge and Ultramafic-Hosted Mineralization: a New Type Of Oceanic Cu-Zn-Co-Au Volcanogenic Massive Sulfide Deposit. Diversity of hydrothermal systems on slow spreading ocean ridges. 2010:321–67.

[46] Thompson G, Mottl M, Rona P. Morphology, mineralogy and chemistry of hydrothermal deposits from the TAG area, 26°N Mid-Atlantic Ridge. Chemical Geology. 1985;49:243–57.

[47] Tivey M, Humphris S, Thompson G, Hannington M, Rona P. Deducing patterns of fluid flow and mixing within the TAG active hydrothermal mound using mineralogical and geochemical data. Journal of Geophysical Research: Solid Earth. 1995;100(B7):12527–55.

[48] Schmale O, Walter M, Schneider von Deimling J, Sültenfuß J, Walker S, Rehder G, et al Fluid and gas fluxes from the Logatchev hydrothermal vent area. Geochemistry, Geophysics, Geosystems. 2012;13:1–12.

[49] Bougault H, Charlou J, Fouquet Y, Needham H, Vaslet N, Appriou P, et al Fast and slow spreading ridges: structure and hydrothermal activity, ultramafic topographic highs, and CH4 output. Journal of Geophysical Research: Solid Earth. 1993;98(B6):9643–51.

[50] Charlou J, Donval J, Konn C, Ondréas H, Fouquet Y, Jean-Baptiste P, et al High production and fluxes of H2 and CH4 and evidence of abiotic hydrocarbon synthesis by serpentinization in ultramafic-hosted hydrothermal systems on the Mid-Atlantic Ridge. Diversity of hydrothermal systems on slow spreading ocean ridges. 2010:265–96.

[51] Petersen J, Zielinski F, Pape T, Seifert R, Moraru C, Amann R, et al Hydrogen is an energy source for hydrothermal vent symbioses. Nature. 2011;476(7359):176–80. 10.1038/nature10325 21833083

[52] Garrity G, Holt J, Huber H, Stetter K, Greene A, Patel B, et al. Deferribacteres phy. nov.. In: Springer, editor. Bergey’s Manual of Systematic Bacteriology New York2001. p. 465–71.

[53] Miroshnichenko M, Slobodkin A, Kostrikina N, L'Haridon S, Nercessian O, Spring S, et al *Deferribacter abyssi* sp. nov., an anaerobic thermophile from deep-sea hydrothermal vents of the Mid-Atlantic Ridge. International journal of systematic and evolutionary microbiology. 2003;53(3):1637–41. 13130062 10.1099/ijs.0.02673-0

[54] Slobodkina G, Kolganova T, Chernyh N, Querellou J, Bonch-Osmolovskaya E, Slobodkin A. *Deferribacter autotrophicus* sp. nov., an iron (III)-reducing bacterium from a deep-sea hydrothermal vent. International journal of systematic and evolutionary microbiology. 2009;59(6):1508–12. 19502344 10.1099/ijs.0.006767-0

[55] Gloter A, Zbinden M, Guyot F, Gaill F, Colliex C. TEM-EELS study of natural ferrihydrite from geological–biological interactions in hydrothermal systems. Earth and Planetary Science Letters. 2004;222(3):947–57.

[56] Mira A, Moran N. Estimating population size and transmission bottlenecks in maternally transmitted endosymbotic bacteria. Microbial Ecology. 2002;44(2):137–43. 10.1007/s00248-002-0012-9 12087426

[57] Thompson J, Higgins D, Gibson T. CLUSTAL W: improving the sensitivity of progressive multiple sequence alignment through sequence weighting, position-specific gap penalties and weight matrix choice. Nucleic acids research. 1994;22(22):4673–80. 7984417 10.1093/nar/22.22.4673 PMC308517

[58] Kumar S, Stecher G, Tamura K. MEGA7: Molecular Evolutionary Genetics Analysis version 7.0 for bigger datasets. Molecular biology and evolution. 2016; msw054. 10.1093/molbev/msw054 PMC8210823 27004904

