# Supplementary figures and images for: Investigation of bacterial communities within the digestive organs of the hydrothermal vent shrimp Rimicaris exoculata provide insights into holobiont geographic clustering
Source: PLoS One. 2017 Mar 15;12(3):e0172543. doi: 10.1371/journal.pone.0172543 (PMC5351989; doi:10.1371/journal.pone.0172543)

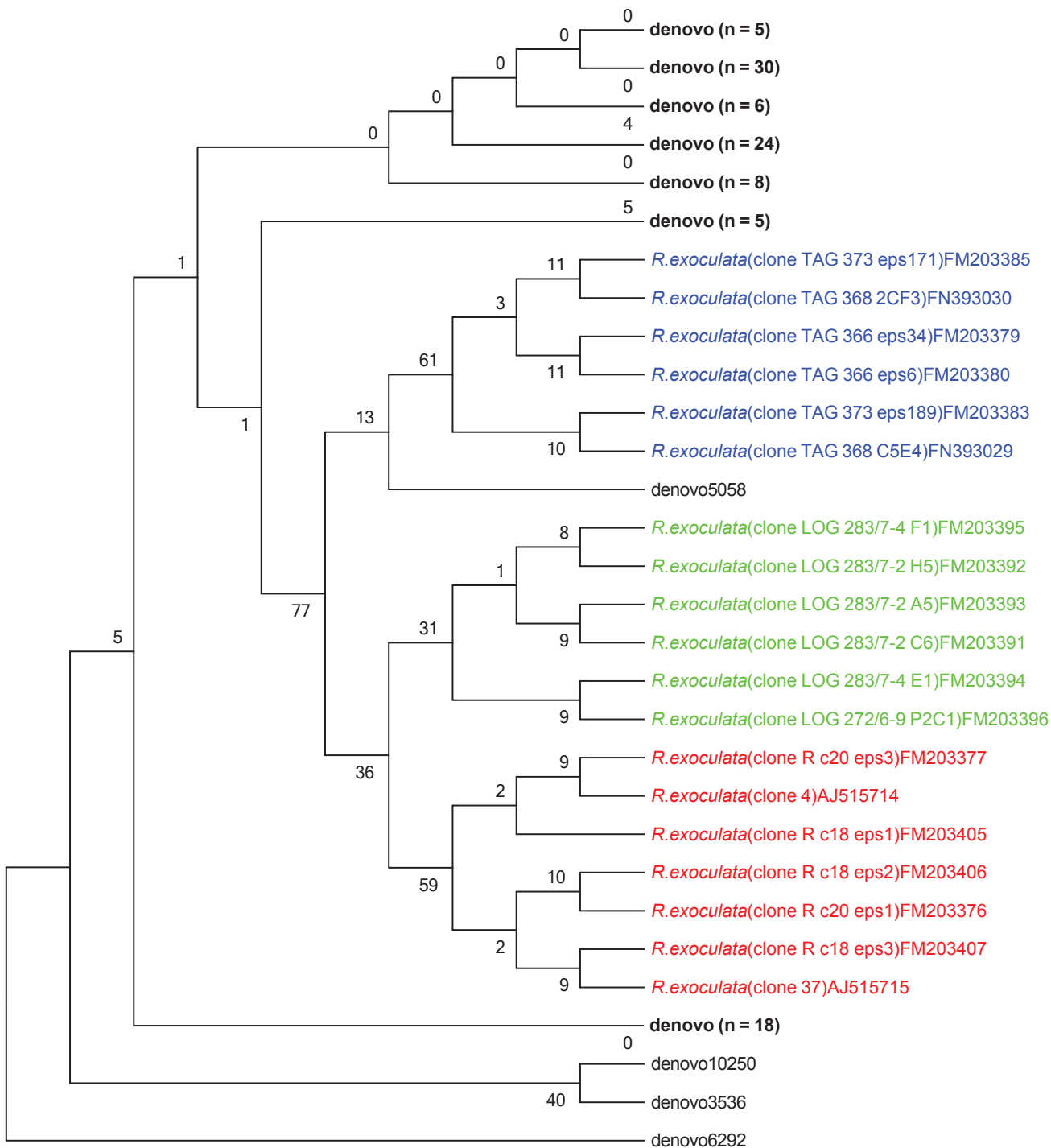

2

Supplement: S1 Fig — Tree illustrates 19 sequences obtained from Durand et al. 2015 [24] and 100 unclassifed bacteria OTUs (denovos), all from the hydrothermal vent shrimp Rimicaris exoculata. Bootstrap support (1000 replicates, Tamura-Nei model) is located either above or below the node. Sequences obtained from Durand et al. 2015, are shown in color (Rainbow = red, TAG = blue, Logatchev = green) and include the clone name and GenBank accession number. Denovo clusters containing multiple sequences were collapsed to reduce the length of the tree. These clusters are shown in bold and entitled “denovo”, followed by the number of sequences contained within the cluster, in parentheses. Scale is measured as number of substitutions per nucleotide site. The alignment was performed using Clustal W [57] in Geneious Pro v5.5.5 (Biomatters Ltd.) and the tree was calculated with the aid of MEGA 7 [58]. (PDF) [file pone.0172543.s001.pdf]

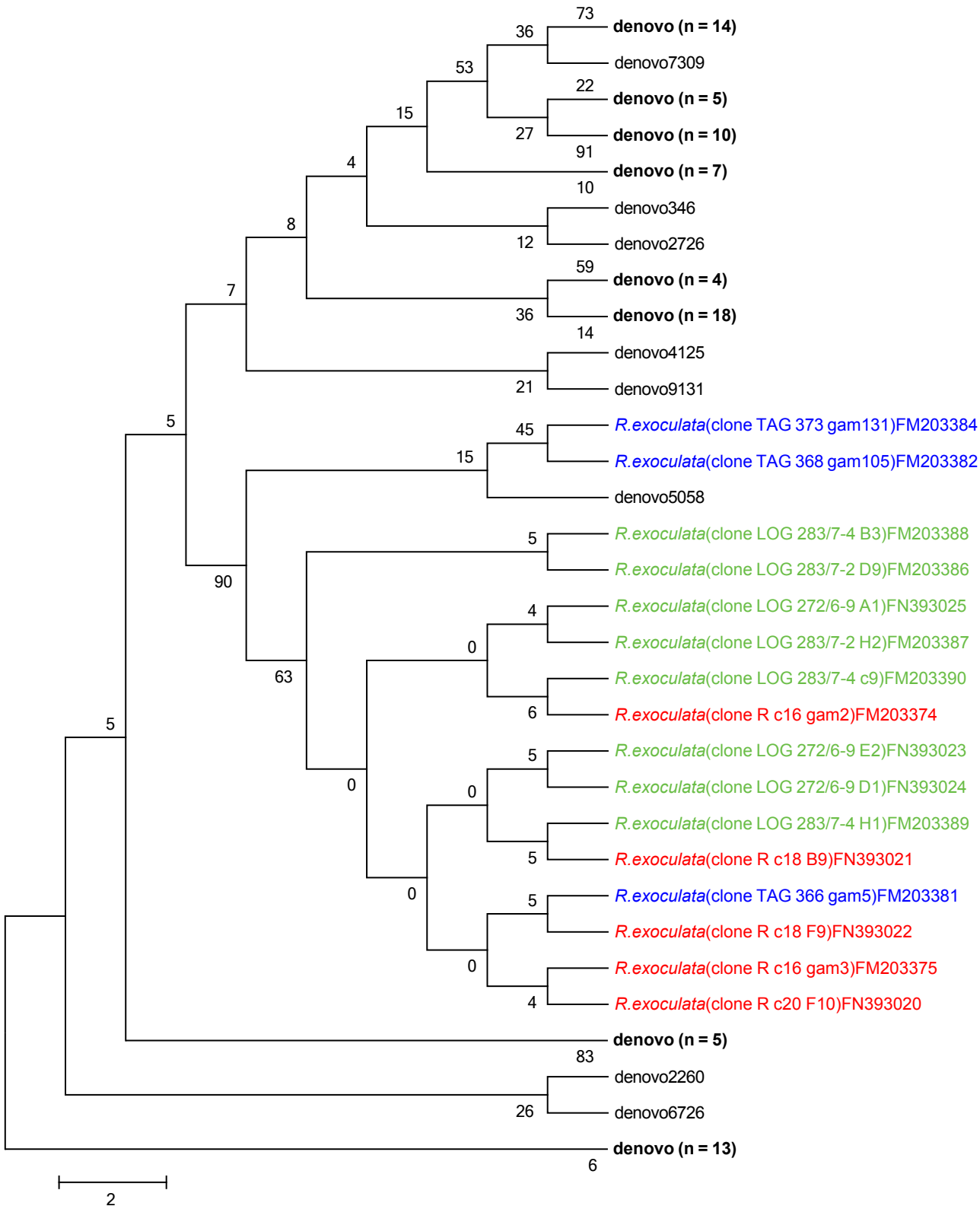

Supplement: S2 Fig — Tree illustrates 16 sequences obtained from Petersen et al. 2010 [14] and 100 unclassifed bacteria OTUs (denovos), all from the hydrothermal vent shrimp Rimicaris exoculata. Bootstrap support (1000 replicates, Tamura-Nei model) is located either above or below the node. Sequences obtained from Petersen et al. 2010, are shown in color (Rainbow = red, TAG = blue, Logatchev = green) and include the clone name and GenBank accession number. Denovo clusters containing multiple sequences were collapsed to reduce the length of the tree. These clusters are shown in bold and entitled “denovo”, followed by the number of sequences contained within the cluster, in parentheses. Scale is measured as number of substitutions per nucleotide site. The alignment was performed using Clustal W [57] in Geneious Pro v5.5.5 (Biomatters Ltd.) and the tree was calculated with the aid of MEGA 7 [58]. (PDF) [file pone.0172543.s002.pdf]

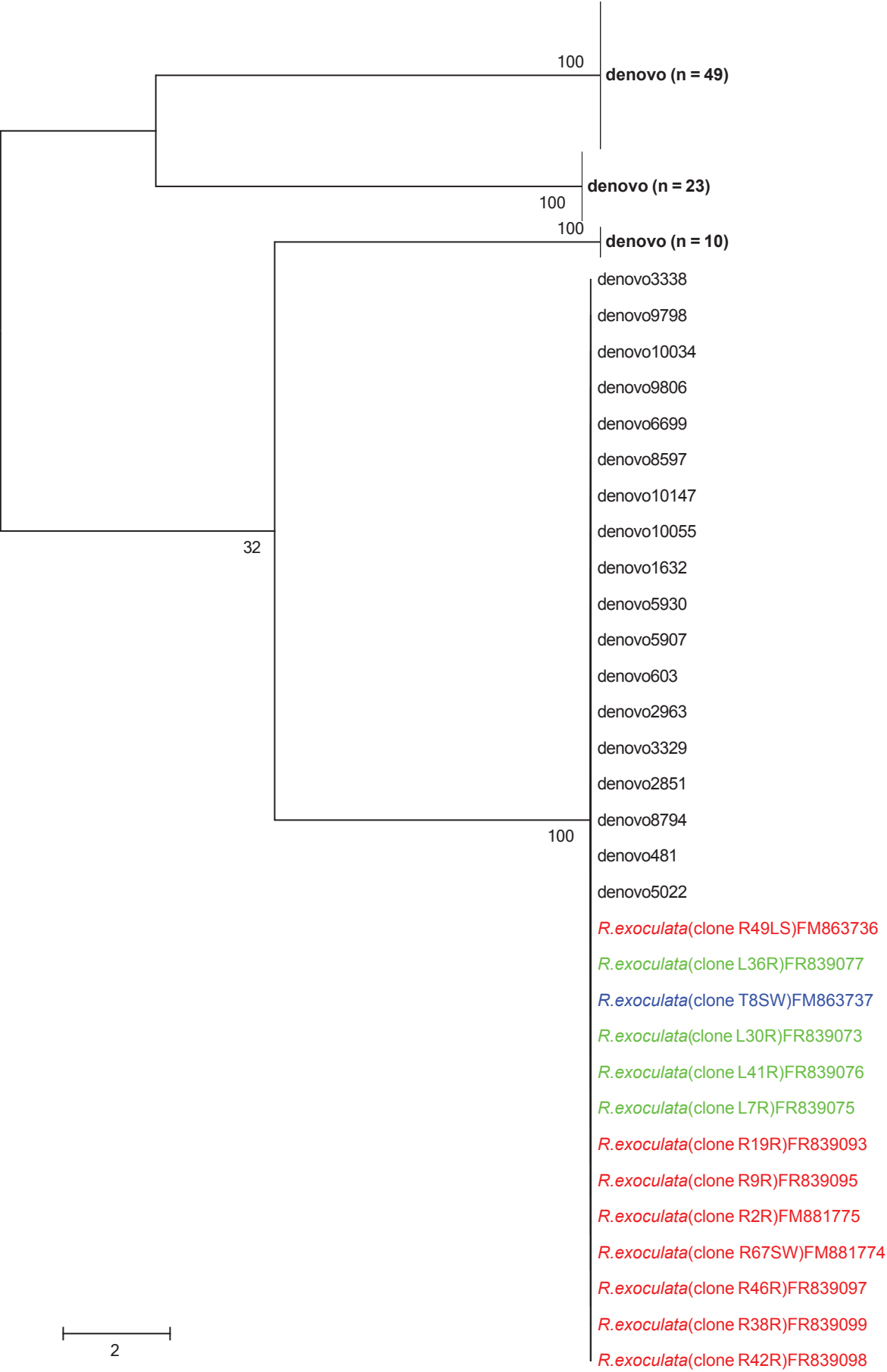

Supplement: S3 Fig — Tree illustrates 13 sequences obtained from Durand et al. 2015 [24] and 100 unclassifed bacteria OTUs (denovos), all from the hydrothermal vent shrimp Rimicaris exoculata. Bootstrap support (1000 replicates, Tamura-Nei model) is located either above or below the node. Sequences obtained from Durand et al. 2015, are shown in color (Rainbow = red, TAG = blue, Logatchev = green) and include the clone name and GenBank accession number. Denovo clusters containing multiple sequences were collapsed to reduce the length of the tree. These clusters are shown in bold and entitled “denovo”, followed by the number of sequences contained within the cluster, in parentheses. Scale is measured as number of substitutions per nucleotide site. The alignment was performed using Clustal W [57] in Geneious Pro v5.5.5 (Biomatters Ltd.) and the tree was calculated with the aid of MEGA 7 [58]. (PDF) [file pone.0172543.s003.pdf]

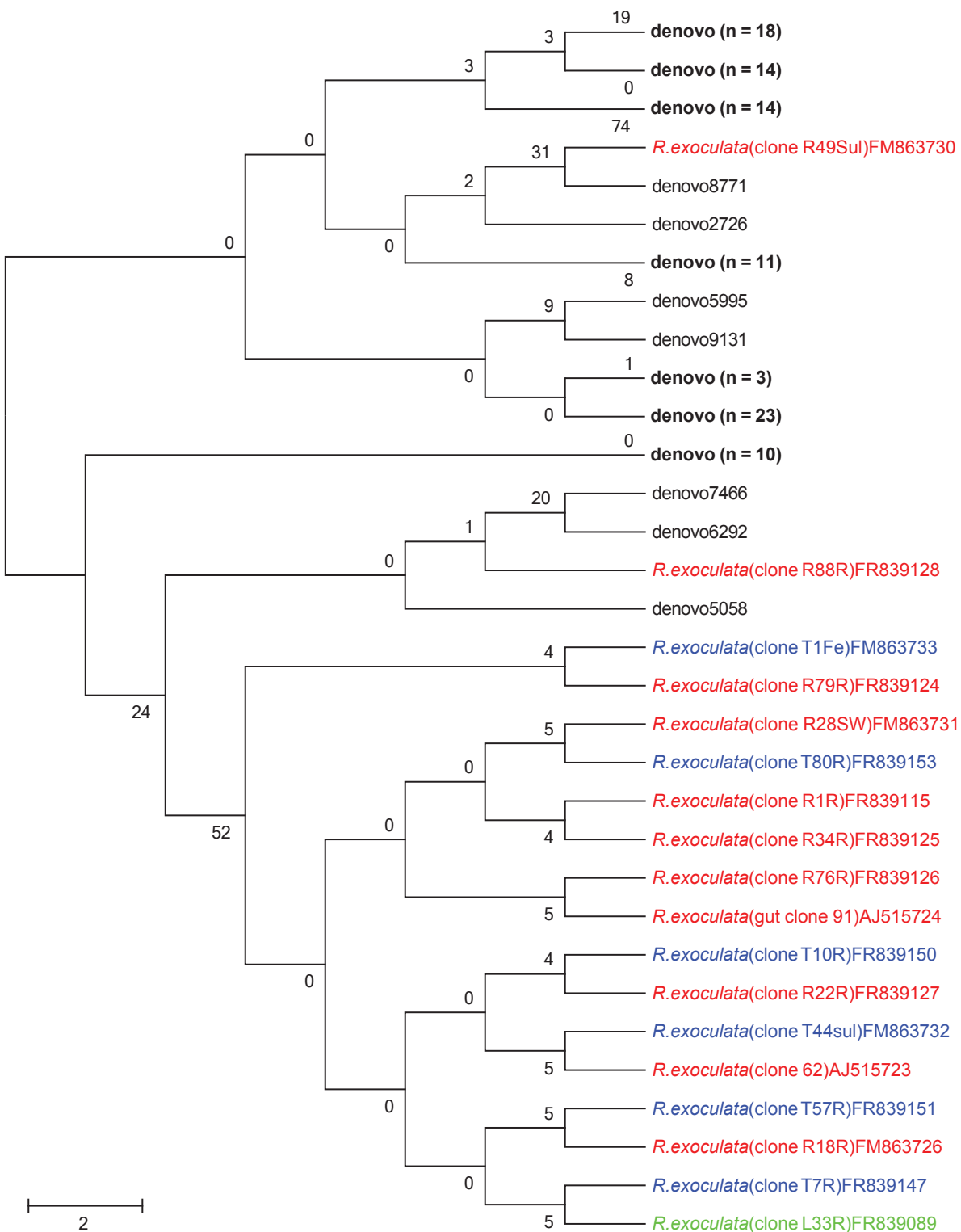

Supplement: S4 Fig — Tree illustrates 18 sequences obtained from Durand et al. 2015 [24] and 100 unclassifed bacteria OTUs (denovos), all from the hydrothermal vent shrimp Rimicaris exoculata. Bootstrap support (1000 replicates, Tamura-Nei model) is located either above or below the node. Sequences obtained from Durand et al. 2015, are shown in color (Rainbow = red, TAG = blue, Logatchev = green) and include the clone name and GenBank accession number. Denovo clusters containing multiple sequences were collapsed to reduce the length of the tree. These clusters are shown in bold and entitled “denovo”, followed by the number of sequences contained within the cluster, in parentheses. Scale is measured as number of substitutions per nucleotide site. The alignment was performed using Clustal W [57] in Geneious Pro v5.5.5 (Biomatters Ltd.) and the tree was calculated with the aid of MEGA 7 [58]. (PDF) [file pone.0172543.s004.pdf]

**A**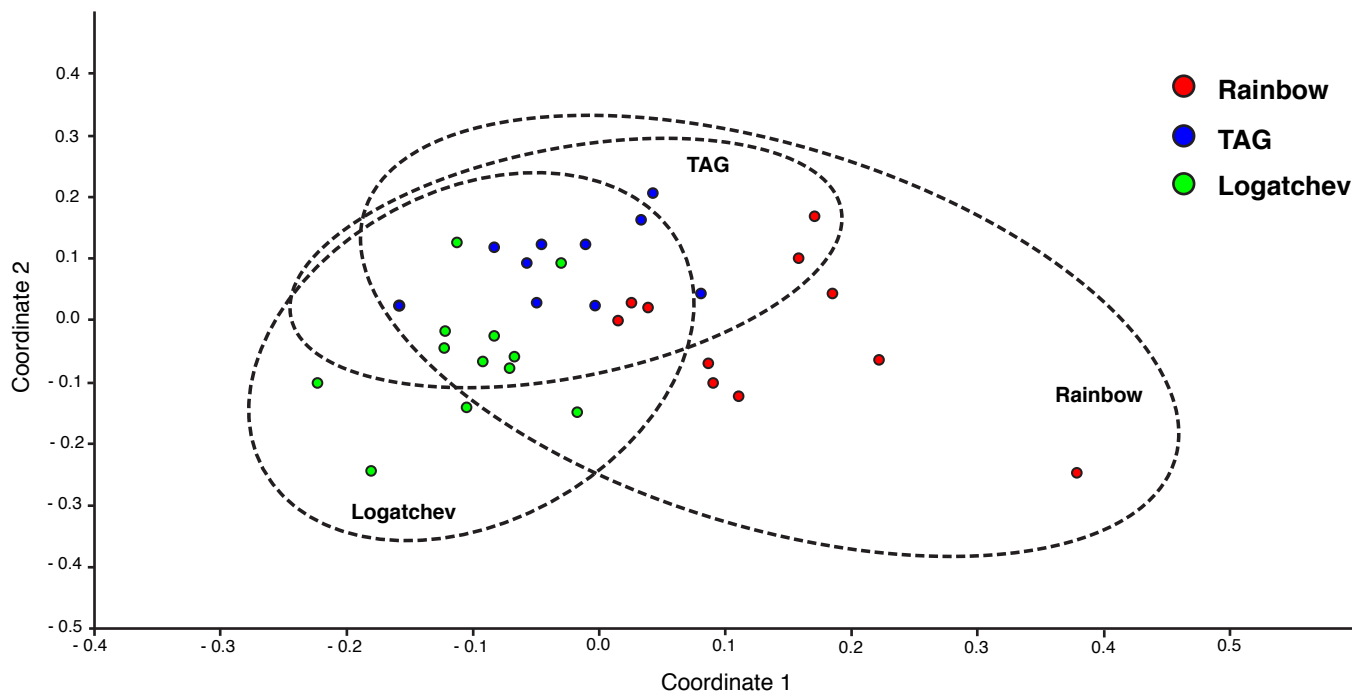**B**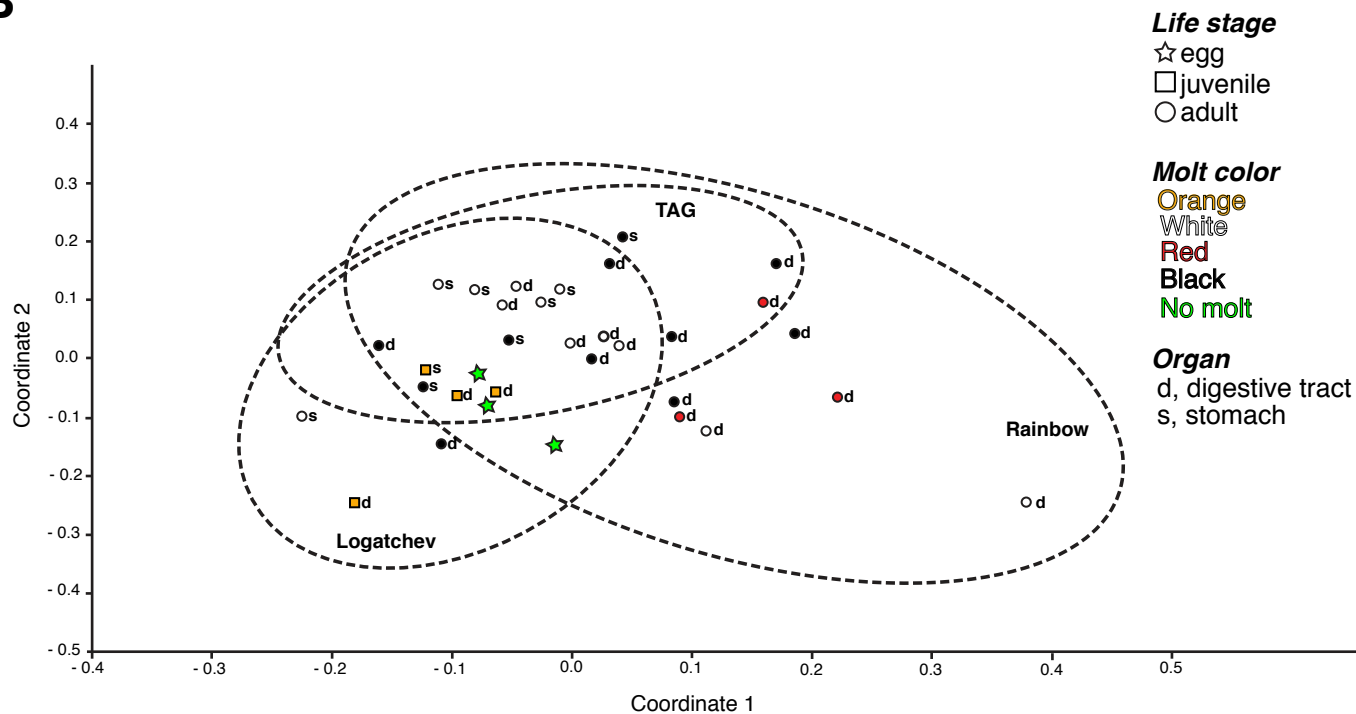

Supplement: S5 Fig — (A) Clustering pattern by vents, with colored circles representing the vent location from where each sample was collected. (B) Clustering pattern by vent, with colored shapes and letters denoting the specific categories for each sample. Each MDS was implemented using Bray-Curtis similarity matrices, calculated in PAST [34]. The 95% concentration ellipses estimate a region where 95% of the population points are expected to fall. “Orange” indicates juveniles, whose carapace is an orange color. Note: the number of samples is 33, rather than 31, as the addition of unidentified bacteria allows us to include two additional samples that have > 500 sequences assigned. (PDF) [file pone.0172543.s005.pdf]

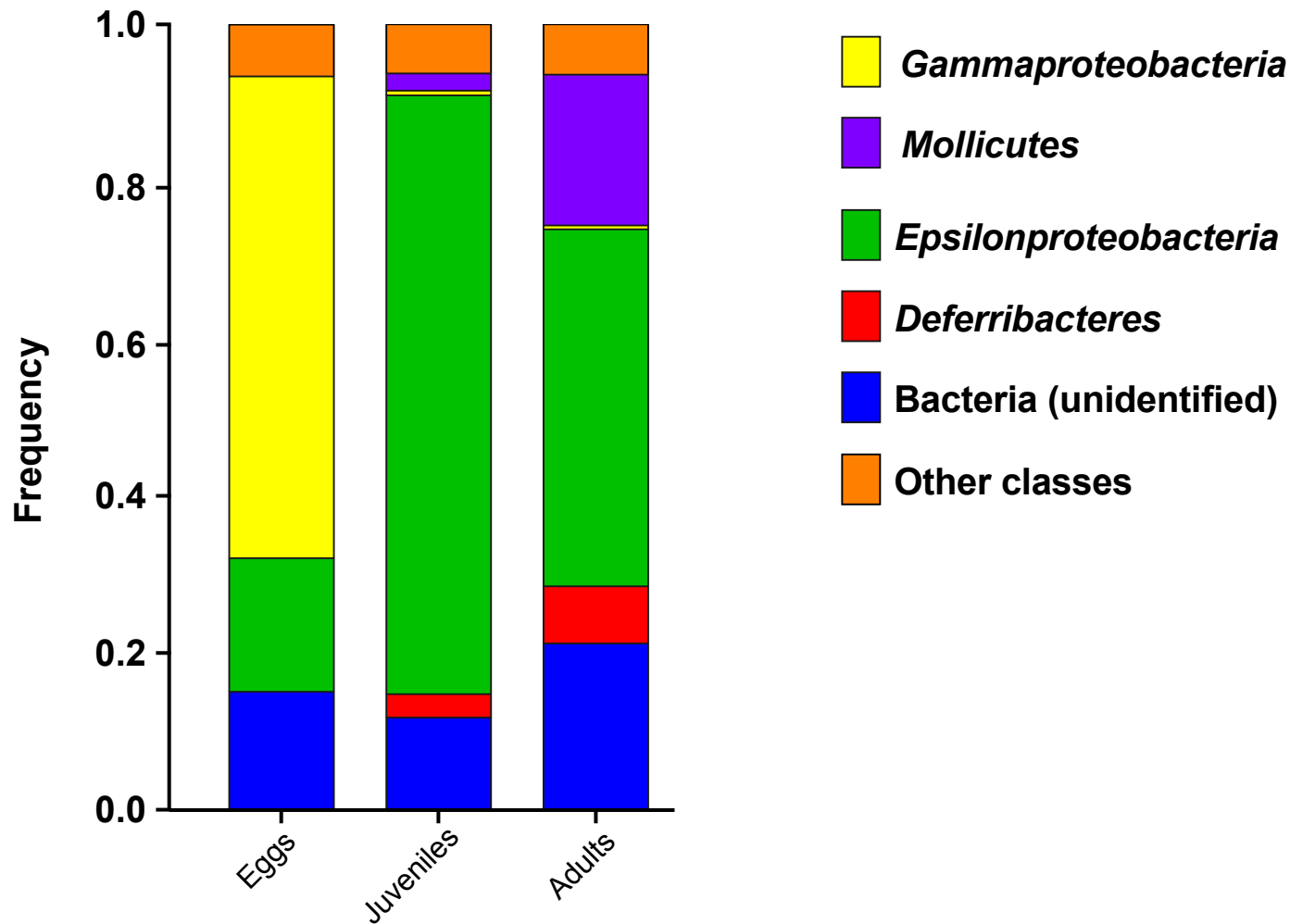

Supplement: S6 Fig — Three main classes are identified across Rimicaris exoculata life stages. “Other classes” contain nine or more less common groups. (PDF) [file pone.0172543.s006.pdf]

**A**

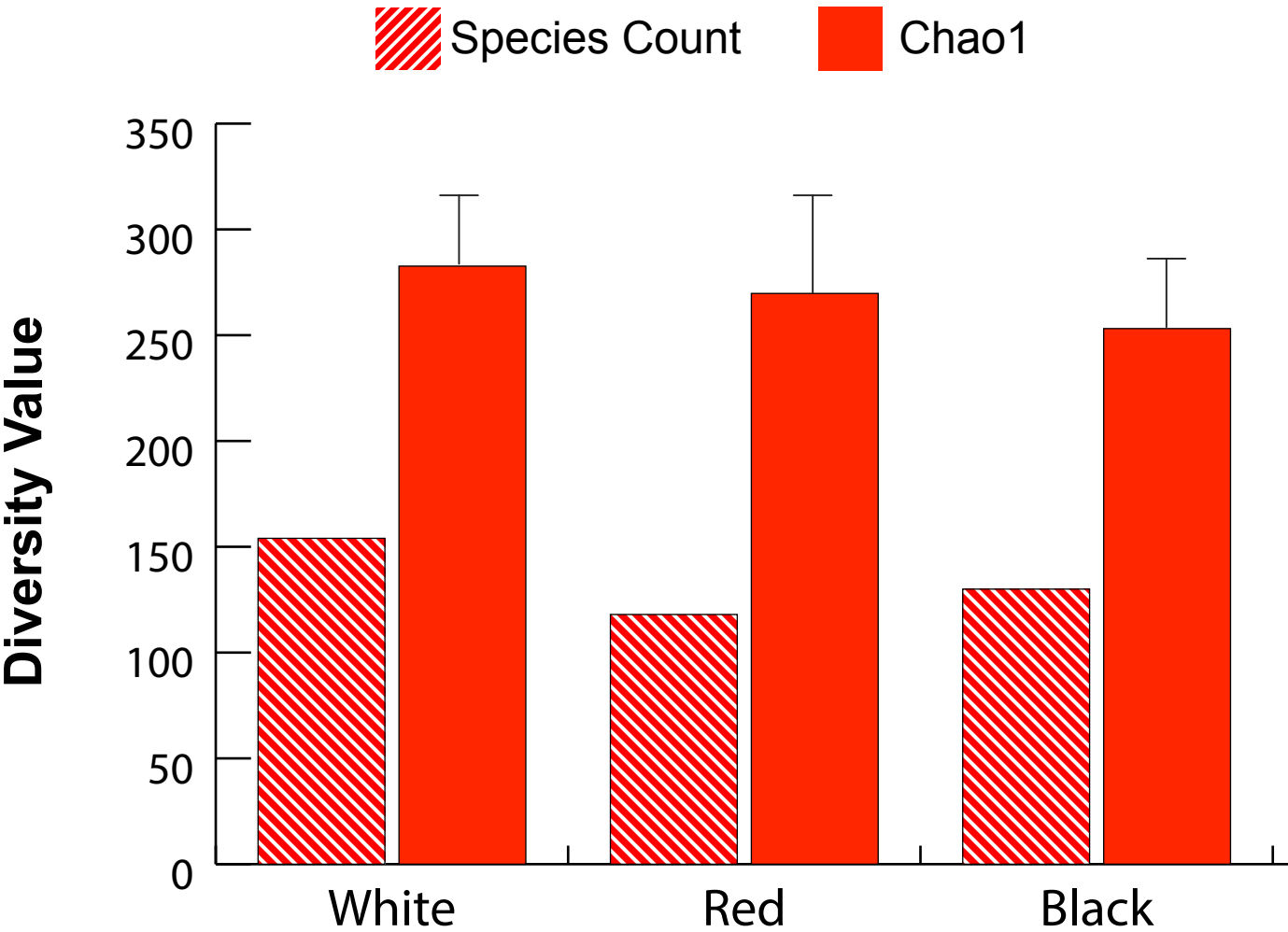

**B**

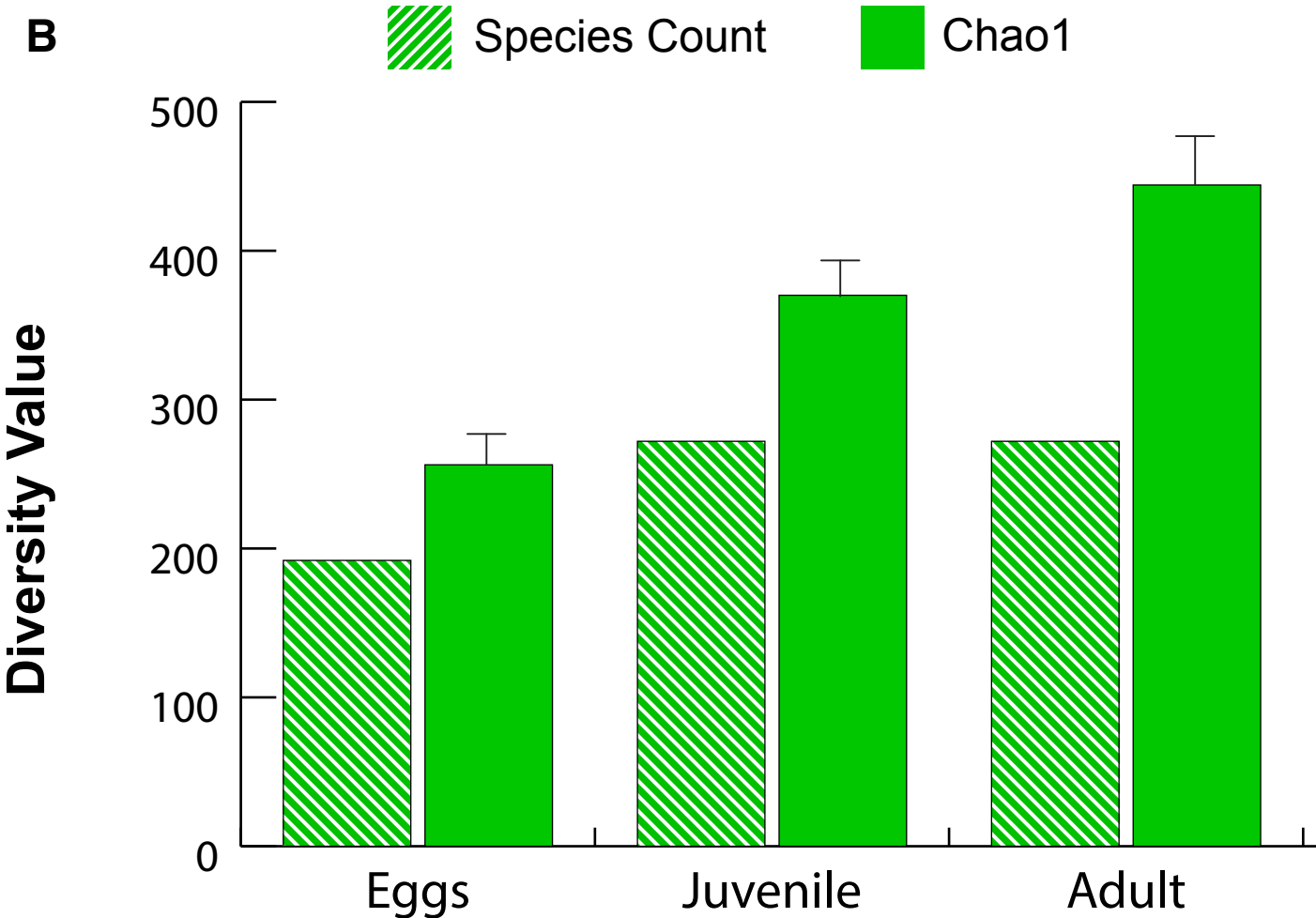

Supplement: S7 Fig — (PDF) [file pone.0172543.s007.pdf]

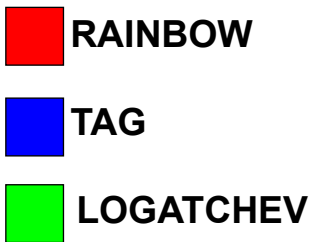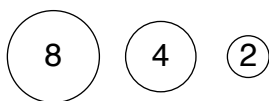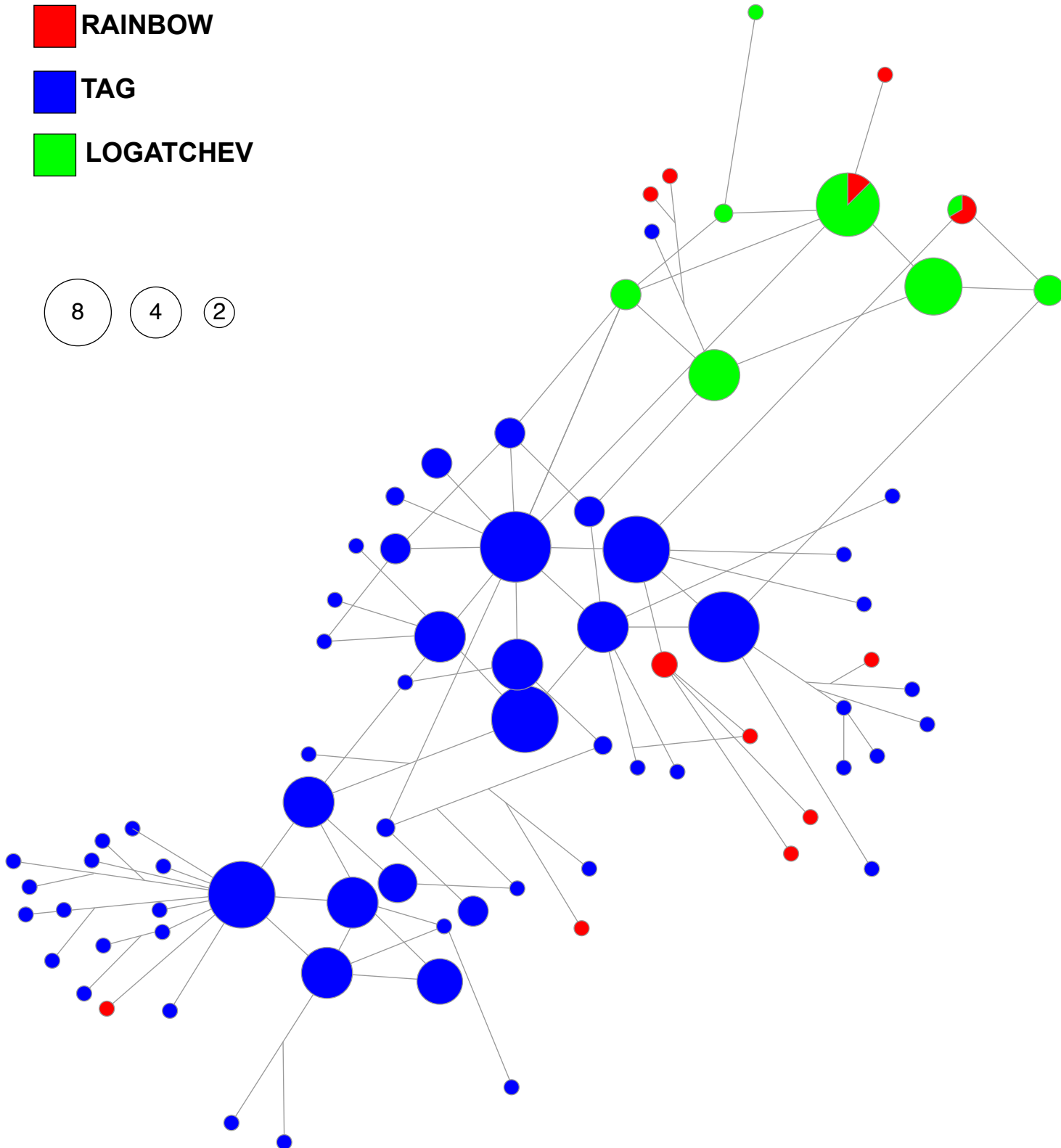

Supplement: S8 Fig — denovo 6909 was composed of 298 sequences. Network was drawn using star-contraction before applying the median-joining calculation to illustrate the clustering of haplotypes; links are not proportional to the number of mutations and therefore, do not illustrate evolutionary divergence between the nodes. (PDF) [file pone.0172543.s008.pdf]
