# Supplementary material for: Investigation of bacterial communities within the digestive organs of the hydrothermal vent shrimp Rimicaris exoculata provide insights into holobiont geographic clustering
Source: PLoS One. 2017 Mar 15;12(3):e0172543. doi: 10.1371/journal.pone.0172543 (PMC5351989; doi:10.1371/journal.pone.0172543)
Supplement: S1 File — (DOCX) [file pone.0172543.s009.docx]

“Investigation of bacterial communities within the digestive organs of the hydrothermal vent shrimp *Rimicaris exoculata* provide insights into holobiont geographic clustering” - Dominique A. Cowart, Lucile Durand, Marie-Anne Cambon-Bonavita, Sophie Arnaud-Haond (corresponding author : [sophie.arnaud@ifremer.fr](mailto:sophie.arnaud@ifremer.fr))

QIIME workflows and commands

For processing samples and assigning taxonomy

1. validate_mapping_file.py (use original 454 fna/qual files and metadata file)

2. split_libraries.py (removes short and low quality sequences, adds barcodes names) 🡪 seqs.fna

3. identify_chimeric_seqs.py (input seqs.fna and 454.fna [to ID chimeras based on parent sequences] files) 🡪 chimeras.txt

4. pick_otus.py (input seqs.fna to pick otus denovo, without reference file) 🡪 seqs_otus.txt

5. pick_rep_set.py (input seqs_otus.txt) 🡪 rep_set.fna

6. assign_taxonomy (input rep_set.fna; reference database: GreenGenes 2013, RDP classifier method) 🡪 rep_set_tax_assignment.txt

7. make_otu_table.py (input rep_set_tax_assign.txt. This otu table includes taxonomic assignments) 🡪 otu_table.biom

8. filter_otus_from_otu_table.py (input otu_table.biom. Remove sequences appearing < 3 times pass –n 3) 🡪 no_singletons.biom

9. filter_otus_from_otu_table.py (input no_singletons.biom and chimeras.txt [step 4]. Removes chimeras) 🡪 no_chimeras.biom

10. convert_biom.py (input no_chimeras.biom. Converts to text file if pass –b –header_key_taxonomy) 🡪 otu_taxa_assign.txt

For Network Analyses

1. filter_fasta.py (input .txt file list of sequence names to filter from seqs.fna [step 2 above]) 🡪 filtered_seqs.fna

2. align_seqs.py (input filtered_seqs.fna to use PYNAST method to align sequences) 🡪 filtered_seqs_aligned.fasta

3. filter_aignment.py (input filtered_seqs_aligned.fasta file to filter alignment) 🡪 filtered_seqs_aligned_pfiltered.fasta

For taxonomic comparisons across phyla and class

1. summarize_taxa_through_plots.py (input otu table no_chimeras.biom [step 9 above] and metadata file [step 1 above] to generate plots based on a category specified in the metadata file. Add –c and name of category to specify separation by that category [i.e. vents, molt size, etc.] 🡪 per_study_otu_tables.biom
