# Supplementary material for: Investigation of bacterial communities within the digestive organs of the hydrothermal vent shrimp Rimicaris exoculata provide insights into holobiont geographic clustering
Source: PLoS One. 2017 Mar 15;12(3):e0172543. doi: 10.1371/journal.pone.0172543 (PMC5351989; doi:10.1371/journal.pone.0172543)
Supplement: S1 Table — (DOCX) [file pone.0172543.s011.docx]

| **Vent** | **Latitude** | **Longitude** | **Depth** | **Year(s)** | **Cruise Campaign** | **No. of samples** |
| --- | --- | --- | --- | --- | --- | --- |
| Rainbow | 36.140 N | 34.000 W | 2200 m | 2005 | EXOMAR | 10 |
|  |  |  |  | 2007 | MoMARDREAM |  |
|  |  |  |  | 2008 | MoMARDREAM |  |
| Trans-Atlantic Geotraverse  (TAG) | 26.039 N | 44.900 W | 3650 m | 2005 | EXOMAR | 9 |
| Logatchev | 14.743 N | 46.593 W | 2860 m | 2007 | SERPENTINE | 12 |
