# Supplementary material for: Investigation of bacterial communities within the digestive organs of the hydrothermal vent shrimp Rimicaris exoculata provide insights into holobiont geographic clustering
Source: PLoS One. 2017 Mar 15;12(3):e0172543. doi: 10.1371/journal.pone.0172543 (PMC5351989; doi:10.1371/journal.pone.0172543)
Supplement: S2 Table — n refers to the number of total samples collected from each vent. Only adults were identified by molt color. (DOCX) [file pone.0172543.s012.docx]

| Categories | | **Rainbow**  *n = 11* | **TAG**  *n = 10* | **Logatchev**  *n = 12* |
| --- | --- | --- | --- | --- |
| **Organ**  (Juveniles and adults only) | Stomach | 0 | 4 | 5 |
|  | Digestive Tract | 10 | 5 | 4 |
| **Molt Color** (Adults only) | White | 4 | 4 | 3 |
|  | Red | 3 | 0 | 0 |
|  | Black | 3 | 5 | 2 |
| **Life Stage** | Eggs | 0 | 0 | 3 |
|  | Juvenile | 0 | 0 | 4 |
|  | Adult | 10 | 9 | 5 |
