# Supplementary material for: Investigation of bacterial communities within the digestive organs of the hydrothermal vent shrimp Rimicaris exoculata provide insights into holobiont geographic clustering
Source: PLoS One. 2017 Mar 15;12(3):e0172543. doi: 10.1371/journal.pone.0172543 (PMC5351989; doi:10.1371/journal.pone.0172543)
Supplement: S3 Table — (DOCX) [file pone.0172543.s013.docx]

| **Sample** | **Sample Name** | **Individual** | **Location** | **Year**  **Collected** | **Depth**  **(m)** | **Coordinates** | **Organs** | **Molt Color** | **Life Stage** |
| --- | --- | --- | --- | --- | --- | --- | --- | --- | --- |
| 1 | 41_RexRTD22 | R22 | Rainbow | 2008 | 2200 | 36.14N 34.00W | Digestive Tract | Red | Adult |
| 2 | 43_RexRTD21 | R21 | Rainbow | 2008 | 2200 | 36.14N 34.00W | Digestive Tract | Red | Adult |
| 3 | 47_RexRTD10 | R10 | Rainbow | 2005 | 2200 | 36.14N 34.00W | Digestive Tract | Black | Adult |
| 4 | 49_RexRTD9 | R9 | Rainbow | 2005 | 2200 | 36.14N 34.00W | Digestive Tract | Black | Adult |
| 5 | 51_RexRTD16 | R16 | Rainbow | 2005 | 2200 | 36.14N 34.00W | Digestive Tract | Black | Adult |
| 6 | 53_RexRTD18 | R18 | Rainbow | 2005 | 2200 | 36.14N 34.00W | Digestive Tract | White | Adult |
| 7 | 61_RexRTD7 | R7 | Rainbow | 2007 | 2200 | 36.14N 34.00W | Digestive Tract | White | Adult |
| 8 | 65_RexRTD19 | R19 | Rainbow | 2008 | 2200 | 36.14N 34.00W | Digestive Tract | White | Adult |
| 9 | 67_RexRTD12 | R12 | Rainbow | 2008 | 2200 | 36.14N 34.00W | Digestive Tract | White | Adult |
| 10 | 87_RexRTD14 | R14 | Rainbow | 2007 | 2200 | 36.14N 34.00W | Digestive Tract | Red | Adult |
| 11 | 69_RexTTD1 | T1 | TAG | 2005 | 3650 | 26.04N 44.90W | Digestive Tract | White | Adult |
| 12 | 70_ RexTE1 | T1 | TAG | 2005 | 3650 | 26.04N 44.90W | Stomach | White | Adult |
| 13 | 72_ RexTTE4 | T4 | TAG | 2005 | 3650 | 26.04N 44.90W | Stomach | White | Adult |
| 14 | 73_ RexTTD2 | T2 | TAG | 2005 | 3650 | 26.04N 44.90W | Digestive Tract | White | Adult |
| 15 | 75_RexTTD12 | T12 | TAG | 2005 | 3650 | 26.04N 44.90W | Digestive Tract | Black | Adult |
| 16 | 76_ RexTE12 | T12 | TAG | 2005 | 3650 | 26.04N 44.90W | Stomach | Black | Adult |
| 17 | 77_ RexTTD11 | T11 | TAG | 2005 | 3650 | 26.04N 44.90W | Digestive Tract | Black | Adult |
| 18 | 78_ RexTE11 | T11 | TAG | 2005 | 3650 | 26.04N 44.90W | Stomach | Black | Adult |
| 19 | 79_ RexTTD9 | T9 | TAG | 2005 | 3650 | 26.04N 44.90W | Digestive Tract | Black | Adult |
| 20 | 15_RexLTD13 | L13 | Logatchev | 2007 | 2860 | 14.74N 46.59W | Digestive Tract | Orange | Juvenile |
| 21 | 17_RexLTD14 | L14 | Logatchev | 2007 | 2860 | 14.74N 46.59W | Digestive Tract | Orange | Juvenile |
| 22 | 19_RexLTD15 | L15 | Logatchev | 2007 | 2860 | 14.74N 46.59W | Digestive Tract | Orange | Juvenile |
| 23 | 21_RexLTD16 | L16 | Logatchev | 2007 | 2860 | 14.74N 46.59W | Digestive Tract | Black | Adult |
| 24 | 22_RexLE16 | L16 | Logatchev | 2007 | 2860 | 14.74N 46.59W | Stomach | Black | Adult |
| 25 | 24_RexLE13 | L13 | Logatchev | 2007 | 2860 | 14.74N 46.59W | Stomach | Orange | Juvenile |
| 26 | 28_RexLE7 | L7 | Logatchev | 2007 | 2860 | 14.74N 46.59W | Stomach | White | Adult |
| 27 | 30_RexLE9 | L9 | Logatchev | 2007 | 2860 | 14.74N 46.59W | Stomach | White | Adult |
| 28 | 32_RexLE10 | L10 | Logatchev | 2007 | 2860 | 14.74N 46.59W | Stomach | White | Adult |
| 29 | 82_RexLW16 | L16 | Logatchev | 2007 | 2860 | 14.74N 46.59W | Eggs | No Molt | Eggs |
| 30 | 84_RexLW2 | L2 | Logatchev | 2007 | 2860 | 14.74N 46.59W | Eggs | No Molt | Eggs |
| 31 | 85_RexLW11 | L11 | Logatchev | 2007 | 2860 | 14.74N 46.59W | Eggs | No Molt | Eggs |
