# Supplementary material for: Investigation of bacterial communities within the digestive organs of the hydrothermal vent shrimp Rimicaris exoculata provide insights into holobiont geographic clustering
Source: PLoS One. 2017 Mar 15;12(3):e0172543. doi: 10.1371/journal.pone.0172543 (PMC5351989; doi:10.1371/journal.pone.0172543)
Supplement: S4 Table — (DOCX) [file pone.0172543.s014.docx]

| **Test** | **Level** | **Vent Location** | **Type** | **Number of Samples** | **Other comments** |
| --- | --- | --- | --- | --- | --- |
| 1  Vent | 2 | All | Rainbow | 10 | DT samples only |
|  |  |  | TAG | 5 |  |
|  |  |  | Logatchev | 4 |  |
| 2A  Organs | 1 | Logatchev | DT | 4 | --- |
|  |  |  | Stomach | 5 |  |
| 2B  Organs | 1 | TAG | DT | 5 | --- |
|  |  |  | Stomach | 4 |  |
| 3  Life Stage | 1 | Logatchev | Eggs | 3 | DT & Stomach |
|  |  |  | Juveniles | 4 |  |
|  |  |  | Adults | 5 |  |
| 4  Molts | 1 | Rainbow | White | 4 | DT samples only |
|  |  |  | Red | 3 |  |
|  |  |  | Black | 3 |  |
