# Supplementary material for: Investigation of bacterial communities within the digestive organs of the hydrothermal vent shrimp Rimicaris exoculata provide insights into holobiont geographic clustering
Source: PLoS One. 2017 Mar 15;12(3):e0172543. doi: 10.1371/journal.pone.0172543 (PMC5351989; doi:10.1371/journal.pone.0172543)
Supplement: S5 Table — Seven samples had > 25% of the OTUs described as ‘Bacteria’; all seven samples were taken from adult R. exoculata. (DOCX) [file pone.0172543.s015.docx]

| **Sample** | **Sample Name** | **Unclassified bacteria (count)** | **Total assigned** | **‘Bacteria’ (%)** |
| --- | --- | --- | --- | --- |
| 1 | 41_RexRTD22 | 146 | 2090 | 6.99 |
| 2 | 43_RexRTD21 | 208 | 1067 | 19.49 |
| 3 | 47_RexRTD10 | 638 | 3109 | 20.52 |
| 4 | 49_RexRTD9 | 320 | 1037 | 30.86 |
| 5 | 51_RexRTD16 | 287 | 2084 | 13.77 |
| 6 | 53_RexRTD18 | 121 | 3280 | 3.69 |
| 7 | 61_RexRTD7 | 94 | 2279 | 4.12 |
| 8 | 65_RexRTD19 | 577 | 1076 | 53.62 |
| 9 | 67_RexRTD12 | 1027 | 4268 | 24.06 |
| 10 | 87_RexRTD14 | 259 | 2608 | 9.93 |
| 11 | 69_RexTTD1 | 909 | 2381 | 38.18 |
| 12 | 70_ RexTE1 | 1112 | 2096 | 53.05 |
| 13 | 72_ RexTTE4 | 464 | 2161 | 21.47 |
| 14 | 73_ RexTTD2 | 1139 | 4808 | 23.69 |
| 15 | 75_RexTTD12 | 299 | 4317 | 6.93 |
| 16 | 76_ RexTE12 | 263 | 1263 | 20.82 |
| 17 | 77_ RexTTD11 | 670 | 14583 | 4.59 |
| 18 | 78_ RexTE11 | 1188 | 5377 | 22.09 |
| 19 | 79_ RexTTD9 | 381 | 888 | 42.91 |
| 20 | 15_RexLTD13 | 179 | 956 | 18.72 |
| 21 | 17_RexLTD14 | 341 | 4537 | 7.52 |
| 22 | 19_RexLTD15 | 311 | 3709 | 8.39 |
| 23 | 21_RexLTD16 | 183 | 1349 | 13.57 |
| 24 | 22_RexLE16 | 503 | 4173 | 12.05 |
| 25 | 24_RexLE13 | 513 | 2212 | 23.19 |
| 26 | 28_RexLE7 | 192 | 2282 | 8.41 |
| 27 | 30_RexLE9 | 882 | 2303 | 38.30 |
| 28 | 32_RexLE10 | 633 | 1180 | 53.64 |
| 29 | 82_RexLW16 | 668 | 2734 | 24.43 |
| 30 | 84_RexLW2 | 446 | 3831 | 11.64 |
| 31 | 85_RexLW11 | 346 | 3134 | 11.04 |
