# Supplementary material for: Investigation of bacterial communities within the digestive organs of the hydrothermal vent shrimp Rimicaris exoculata provide insights into holobiont geographic clustering
Source: PLoS One. 2017 Mar 15;12(3):e0172543. doi: 10.1371/journal.pone.0172543 (PMC5351989; doi:10.1371/journal.pone.0172543)
Supplement: S6 Table — The Bray-Curtis similarity index at 9999 permutations was used (*) defines significant relationships at p = 0.05. (DOCX) [file pone.0172543.s016.docx]

| **All vents** | | | | **ANOSIM** | | |
| --- | --- | --- | --- | --- | --- | --- |
| *Ho: no difference between vents* | | | | *p = 0.0003*, R = 0.634* | | |
| **Logatchev** | | | |  | | |
| *Ho: no difference between digestive tract and stomach* | | | | *p = 0.071, R = 0.244* | | |
| *Ho: no difference between various life stages* | | | | *p* = 0.005*, *R* = 0.382 | | |
| **TAG** | | | |  | | |
| Ho: *no difference between digestive tract and stomach* | | | | *p = 0.249, R = 0.111* | | |
| **Rainbow** | | | |  | | |
| *Ho: no difference between white, red and black molts* | | | | *p = 0.656, R = 0.051* | | |
| **Vents** | | | | | | |
|  | Rainbow | | TAG | | | Logatchev |
| Rainbow | --- | | 0.0013‡ | | | 0.0023‡ |
| TAG | 0.0013‡ | | --- | | | 0.2009 |
| Logatchev | 0.0023‡ | | 0.2009 | | | --- |
|  | | | | | | |
| **Life Stage (Logatchev)** | | | | | | |
|  | Eggs | | Juveniles | | | Adults |
| Eggs | --- | | 0.025* | | | 0.018* |
| Juveniles | 0.025* | | --- | | | 0.330 |
| Adults | 0.018* | | 0.330 | | | --- |
|  | | | | | | |
| **Molts (Rainbow)** | | | | | | |
|  | White | | Red | | | Black |
| White | --- | | 0.863 | | | 0.256 |
| Red | 0.863 | | --- | | | 0.863 |
| Black | 0.256 | |  | | | --- |
|  | | | | | | |
| **By Organs (TAG)** | | | | | | |
|  | | Digestive Tract | | | Stomach | |
| Digestive Tract | | --- | | | 0.975 | |
| Stomach | | 0.975 | | | --- | |
|  | |  | | |  | |
| **By Organs (Logatchev)** | | | | | | |
|  | | Digestive Tract | | | Stomach | |
| Digestive Tract | | --- | | | 0.070 | |
| Stomach | | 0.070 | | | --- | |

* Rainbow is different from other vents

‡ eggs are different from other life stages
