# Supplementary material for: Investigation of bacterial communities within the digestive organs of the hydrothermal vent shrimp Rimicaris exoculata provide insights into holobiont geographic clustering
Source: PLoS One. 2017 Mar 15;12(3):e0172543. doi: 10.1371/journal.pone.0172543 (PMC5351989; doi:10.1371/journal.pone.0172543)
Supplement: S7 Table — (DOCX) [file pone.0172543.s017.docx]

| Vent | Group | | Species Count | Chao1 ± SE |
| --- | --- | --- | --- | --- |
| **Rainbow** | Organ | Stomach | n/a | n/a |
|  |  | Digestive Tract | 895 | 1377.0 ± 58.8 |
|  | Molt | White | 525 |  |
|  |  | Red | 350 | 918.0 ± 108.6 |
|  |  | Black | 535 | 1322.6 ± 119.7 |
|  | Life Stage | Eggs | n/a | n/a |
|  |  | Juvenile | n/a | n/a |
|  |  | Adult | 895 | 1377.0 ± 58.8 |
| **TAG** | Organ | Stomach | 774 | 1566.4 ± 97.1 |
|  |  | Digestive Tract | 1066 | 1964.7 ± 93.9 |
|  | Molt | White | 851 | 1349.2 ± 60.3 |
|  |  | Red | n/a | n/a |
|  |  | Black | 873 | 1503.3 ± 72.5 |
|  | Life Stage | Eggs | n/a | n/a |
|  |  | Juvenile | n/a | n/a |
|  |  | Adult | 1242 | 1609.2 ± 40.93 |
| **Logatchev** | Organ | Stomach | 857 | 1360.0 ± 59.8 |
|  |  | Digestive Tract | 609 | 1043.1 ± 60.3 |
|  | Molt | White | 597 | 1207.7 ± 83.8 |
|  |  | Red | n/a | n/a |
|  |  | Black | 438 | 894.25 ± 74.7 |
|  | Life Stage | Eggs | 556 | 902.4 ± 50.6 |
|  |  | Juvenile | 693 | 1247.4 ± 73.0 |
|  |  | Adult | 811 | 1396.2 ± 70.4 |
