# Supplementary material for: Investigation of bacterial communities within the digestive organs of the hydrothermal vent shrimp Rimicaris exoculata provide insights into holobiont geographic clustering
Source: PLoS One. 2017 Mar 15;12(3):e0172543. doi: 10.1371/journal.pone.0172543 (PMC5351989; doi:10.1371/journal.pone.0172543)
Supplement: S8 Table — *asterisk denotes less represented phyla that include classes of Acidobacteria, Actinobacteria, Bacteroidetes, Chlorobi, Cyanobacteria, Firmicutes, GB02, SR1 and other proteobacteria. (DOCX) [file pone.0172543.s018.docx]

| **Rainbow** | | | | | | | | |
| --- | --- | --- | --- | --- | --- | --- | --- | --- |
| Class | Organ | | Molt Color | | | Life Stage | | |
|  | Digestive Tract | | White | Red | Black | Eggs | Juveniles | Adult |
| Unidentified Bacteria | 0.167 | | 0.167 | 0.106 | 0.220 |  |  | 0.167 |
| *Deferribacteres* | 0.715 | | 0.753 | 0.819 | 0.564 | --- | --- | 0.715 |
| *Epsilonproteobacteria* | 0.052 | | 0.041 | 0.054 | 0.067 | --- | --- | 0.052 |
| *Mollicutes* | 0.049 | | 0.020 | 0.007 | 0.133 | --- | --- | 0.049 |
| *Gammaproteobacteria* | 0.002 | | 0.001 | 0.003 | 0.002 | --- | --- | 0.002 |
| Others* | 0.015 | | 0.018 | 0.011 | 0.014 | --- | --- | 0.015 |
| **TAG** | | | | | | | | |
| Class | Organs | | | Molt Color | | Life Stage | | |
|  | Stomach | Digestive Tract | | White | Black | --- | --- | Adult |
| Unidentified Bacteria | 0.278 | 0.153 | | 0.359 | 0.106 |  |  | 0.188 |
| *Deferribacteres* | 0.003 | 0.120 | | 0.205 | 0.031 | --- | --- | 0.088 |
| *Epsilonproteobacteria* | 0.180 | 0.381 | | 0.015 | 0.473 | --- | --- | 0.325 |
| *Mollicutes* | 0.418 | 0.116 | | 0.385 | 0.112 | --- | --- | 0.200 |
| *Gammaproteobacteria* | 0.015 | 0.017 | | 0.001 | 0.023 | --- | --- | 0.016 |
| Others* | 0.106 | 0.213 | | 0.035 | 0.255 | --- | --- | 0.183 |
| **Logatchev** | | | | | | | | |
| Class | Organ | | | Molt Color | | Life Stage | | |
|  | Stomach | Digestive Tract | | White | Black | Eggs | Juveniles | Adult |
| Unidentified Bacteria | 0.224 | 0.096 | | 0.234 | 0.211 | 0.151 | 0.118 | 0.212 |
| *Deferribacteres* | 0.001 | 0.109 | | 0.002 | 0.086 | 0.000 | 0.030 | 0.073 |
| *Epsilonproteobacteria* | 0.504 | 0.731 | | 0.393 | 0.448 | 0.170 | 0.762 | 0.455 |
| *Mollicutes* | 0.189 | 0.012 | | 0.345 | 0.062 | 0.000 | 0.022 | 0.192 |
| *Gammaproteobacteria* | 0.005 | 0.006 | | 0.002 | 0.105 | 0.613 | 0.006 | 0.005 |
| Others* | 0.077 | 0.046 | | 0.024 | 0.088 | 0.066 | 0.062 | 0.063 |
