# Supplementary material for: Investigation of bacterial communities within the digestive organs of the hydrothermal vent shrimp Rimicaris exoculata provide insights into holobiont geographic clustering
Source: PLoS One. 2017 Mar 15;12(3):e0172543. doi: 10.1371/journal.pone.0172543 (PMC5351989; doi:10.1371/journal.pone.0172543)
Supplement: S9 Table — The Bray-Curtis similarity index at 9999 permutations was used (*) defines significant relationships at p = 0.05. (DOCX) [file pone.0172543.s019.docx]

| **Vents** | | | | | | | | | | | | | | | | | |
| --- | --- | --- | --- | --- | --- | --- | --- | --- | --- | --- | --- | --- | --- | --- | --- | --- | --- |
| ***ANOSIM*** | | | | | | | | | ***PERMANOVA*** | | | | | | | | |
| p value | | | | | | | 0.0004* | | p value | | | | | | | 0.0002* | |
| R value | | | | | | | 0.630 | | F value | | | | | | | 4.212 | |
| Mean rank within | | | | | | | 51.330 | | Total sum of squares | | | | | | | 5.166 | |
| Mean rank between | | | | | | | 105.20 | | Within group sum of squares | | | | | | | 3.384 | |
| *Pairwise p values* | | | | | | | | | *Pairwise p values* | | | | | | | | |
|  | | TAG | | Logatchev | | | Rainbow | |  | | TAG | | Logatchev | | | Rainbow | |
| TAG | |  | | 0.245 | | | 0.001* | | TAG | |  | | 0.109 | | | 0.001* | |
| Logatchev | | 0.245 | |  | | | 0.002* | | Logatchev | | 0.109 | |  | | | 0.002* | |
| Rainbow | | 0.001* | | 0.002* | | |  | | Rainbow | | 0.001* | | 0.002* | | |  | |
| **Logatchev (by organ)** | | | | | | | | | | | | | | | | | |
| ***ANOSIM*** | | | | | | | | | ***PERMANOVA*** | | | | | | | | |
| p value | | | | | | | 0.066 | | p value | | | | | | | 0.117 | |
| R value | | | | | | | 0.288 | | F value | | | | | | | 1.842 | |
| Mean rank within | | | | | | | 15.630 | | Total sum of squares | | | | | | | 2.439 | |
| Mean rank between | | | | | | | 20.800 | | Within group sum of squares | | | | | | | 1.931 | |
| *Pairwise p values* | | | | | | | | | *Pairwise p values* | | | | | | | | |
|  | | | Digestive Tract | | | Stomach | | |  | | | Digestive Tract | | | Stomach | | |
| Digestive Tract | | |  | | | 0.062 | | | Digestive Tract | | |  | | | 0.118 | | |
| Stomach | | | 0.062 | | |  | | | Stomach | | | 0.118 | | |  | | |
| **TAG (by organ)** | | | | | | | | | | | | | | | | | |
| ***ANOSIM*** | | | | | | | | | ***PERMANOVA*** | | | | | | | | |
| p value | | | | | | 0.974 | | | p value | | | | | | 0.562 | | |
| R value | | | | | | -0.112 | | | F value | | | | | | 0.818 | | |
| Mean rank within | | | | | | 19.630 | | | Total sum of squares | | | | | | 2.373 | | |
| Mean rank between | | | | | | 17.600 | | | Within group sum of squares | | | | | | 2.125 | | |
| *Pairwise p values* | | | | | | | | | *Pairwise p values* | | | | | | | | |
|  | | | Digestive Tract | | | Stomach | | |  | | | Digestive Tract | | | Stomach | | |
| Digestive Tract | | |  | | | 0.975 | | | Digestive Tract | | |  | | | 0.556 | | |
| Stomach | | | 0.975 | | |  | | | Stomach | | | 0.556 | | |  | | |
| **Logatchev (by Life Stage)** | | | | | | | | | | | | | | | | | |
| ***ANOSIM*** | | | | | | | | | ***PERMANOVA*** | | | | | | | | |
| p value | | | | | | | 0.011* | | p value | | | | | | | 0.003* | |
| R value | | | | | | | 0.373 | | F value | | | | | | | 2.944 | |
| Mean rank within | | | | | | | 24.740 | | Total sum of squares | | | | | | | 3.606 | |
| Mean rank between | | | | | | | 37.040 | | Within group sum of squares | | | | | | | 2.180 | |
| *Pairwise p values* | | | | | | | | | *Pairwise p values* | | | | | | | | |
|  | | | Eggs | | Juveniles | | | Adults |  | | | Eggs | | Juveniles | | | Adults |
| Eggs | | |  | | 0.029* | | | 0.053 | Eggs | | |  | | 0.029* | | | 0.017* |
| Juveniles | | | 0.029* | |  | | | 0.295 | Juveniles | | | 0.029* | |  | | | 0.180 |
| Adults | | | 0.053 | | 0.295 | | |  | Adults | | | 0.017* | | 0.180 | | |  |
| **Rainbow (by Molt Color)** | | | | | | | | | | | | | | | | | |
| ***ANOSIM*** | | | | | | | | | ***PERMANOVA*** | | | | | | | | |
| p value | | | | | | | 0.606 | | p value | | | | | | | 0.684 | |
| R value | | | | | | | -0.066 | | F value | | | | | | | 0.670 | |
| Mean rank within | | | | | | | 24.080 | | Total sum of squares | | | | | | | 0.980 | |
| Mean rank between | | | | | | | 22.610 | | Within group sum of squares | | | | | | | 0.670 | |
| *Pairwise p values* | | | | | | | | | *Pairwise p values* | | | | | | | | |
|  | White | | | Red | | | Black | |  | White | | | Red | | | Black | |
| White |  | | | 0.826 | | | 0.293 | | White |  | | | 0.630 | | | 0.461 | |
| Red | 0.826 | | |  | | | 0.799 | | Red | 0.630 | | |  | | | 0.5956 | |
| Black | 0.293 | | | 0.799 | | |  | | Black | 0.461 | | | 0.596 | | |  | |
