# Supplementary material for: Investigation of bacterial communities within the digestive organs of the hydrothermal vent shrimp Rimicaris exoculata provide insights into holobiont geographic clustering
Source: PLoS One. 2017 Mar 15;12(3):e0172543. doi: 10.1371/journal.pone.0172543 (PMC5351989; doi:10.1371/journal.pone.0172543)
Supplement: S10 Table — *asterisk denotes less represented phyla that include classes of Acidobacteria, Actinobacteria, Bacteroidetes, Chlorobi, Cyanobacteria, Firmicutes, GB02, SR1 and other proteobacteria. (DOCX) [file pone.0172543.s020.docx]

| **Rainbow** | | | | | | | | |
| --- | --- | --- | --- | --- | --- | --- | --- | --- |
| Class | Organ | | Molt Color | | | Life Stage | | |
|  | Digestive Tract | | White | Red | Black | Eggs | Juveniles | Adult |
| *Deferribacteres* | 0.858 | | 0.904 | 0.916 | 0.713 | --- | --- | 0.858 |
| *Epsilonproteobacteria* | 0.062 | | 0.048 | 0.061 | 0.088 | --- | --- | 0.062 |
| *Mollicutes* | 0.060 | | 0.024 | 0.007 | 0.179 | --- | --- | 0.060 |
| *Gammaproteobacteria* | 0.002 | | 0.001 | 0.003 | 0.002 | --- | --- | 0.002 |
| Others* | 0.018 | | 0.022 | 0.013 | 0.018 | --- | --- | 0.018 |
| **TAG** | | | | | | | | |
| Class | Organs | | | Molt Color | | Life Stage | | |
|  | Stomach | Digestive Tract | | White | Black | --- | --- | Adult |
| *Deferribacteres* | 0.004 | 0.143 | | 0.330 | 0.035 | --- | --- | 0.108 |
| *Epsilonproteobacteria* | 0.249 | 0.455 | | 0.023 | 0.529 | --- | --- | 0.403 |
| *Mollicutes* | 0.579 | 0.133 | | 0.605 | 0.125 | --- | --- | 0.245 |
| *Gammaproteobacteria* | 0.021 | 0.020 | | 0.002 | 0.026 | --- | --- | 0.020 |
| Others* | 0.148 | 0.250 | | 0.040 | 0.285 | --- | --- | 0.224 |
| **Logatchev** | | | | | | | | |
| Class | Organ | | | Molt Color | | Life Stage | | |
|  | Stomach | Digestive Tract | | White | Black | Eggs | Juveniles | Adult |
| *Deferribacteres* | 0.001 | 0.121 | | 0.002 | 0.168 | <0.001 | 0.034 | 0.092 |
| *Epsilonproteobacteria* | 0.649 | 0.808 | | 0.452 | 0.681 | 0.200 | 0.864 | 0.577 |
| *Mollicutes* | 0.243 | 0.013 | | 0.513 | 0.017 | <0.001 | 0.025 | 0.244 |
| *Gammaproteobacteria* | 0.006 | 0.007 | | 0.002 | 0.010 | 0.722 | 0.007 | 0.006 |
| Others* | 0.100 | 0.051 | | 0.030 | 0.124 | 0.078 | 0.071 | 0.081 |
