# Supplementary material for: Investigation of bacterial communities within the digestive organs of the hydrothermal vent shrimp Rimicaris exoculata provide insights into holobiont geographic clustering
Source: PLoS One. 2017 Mar 15;12(3):e0172543. doi: 10.1371/journal.pone.0172543 (PMC5351989; doi:10.1371/journal.pone.0172543)
Supplement: S11 Table — Most common haplotypes are defined as those represented by ≥20 sequences within common operational taxonomic units (OTU) for which networks were not drawn. Each common haplotype is listed by name, number of sequences within each haplotype, and the percentage of sequences from each vent. (DOCX) [file pone.0172543.s021.docx]

| **Denovo6700**  *(Deferribacteres)*  Total number of sequences: 19,734  Total number of haplotypes: 1,128 | | | | |
| --- | --- | --- | --- | --- |
| **Haplotypes** | | **Percentages** | | |
| Name | Number of sequences | Rainbow | TAG | Logatchev |
| HAP_1 | 15,265 | 87.3 | 8.3 | 4.4 |
| HAP_2 | 1,106 | 0 | 99 | 1 |
| HAP_5 | 20 | 80 | 10 | 10 |
| HAP_8 | 21 | 90.5 | 0 | 9.5 |
| HAP_14 | 30 | 96.6 | 0 | 3.4 |
| HAP_31 | 24 | 87.5 | 4.2 | 8.3 |
| HAP_32 | 84 | 83.2 | 12 | 4.8 |
| HAP_38 | 25 | 92 | 4 | 4 |
| HAP_44 | 25 | 88 | 8 | 4 |
| HAP_48 | 25 | 100 | 0 | 0 |
| HAP_51 | 29 | 82.7 | 7 | 10.3 |
| HAP_52 | 21 | 95.2 | 0 | 4.8 |
| HAP_53 | 20 | 90 | 0 | 10 |
| HAP_55 | 21 | 90.6 | 4.7 | 4.7 |
| HAP_58 | 27 | 96.3 | 0 | 3.7 |
| HAP_60 | 38 | 97.4 | 2.6 | 0 |
| HAP_65 | 24 | 87.5 | 4.2 | 8.3 |
| HAP_78 | 22 | 77.3 | 13.6 | 9.1 |
| HAP_80 | 30 | 93.4 | 3.3 | 3.3 |
| HAP_103 | 29 | 89.6 | 3.4 | 7 |
| HAP_106 | 39 | 79.5 | 15.4 | 5.1 |
| HAP_115 | 23 | 87 | 8.7 | 4.3 |
| HAP_118 | 23 | 91.3 | 0 | 8.7 |
| HAP_119 | 21 | 86 | 14 | 0 |
| HAP_120 | 28 | 68 | 14 | 18 |
| HAP_127 | 26 | 69.2 | 15.4 | 15.4 |
| HAP_131 | 24 | 91.6 | 4.2 | 4.2 |
| HAP_150 | 31 | 93.6 | 3.2 | 3.2 |
| HAP_160 | 88 | 42 | 2.3 | 55.7 |
| HAP_170 | 24 | 75 | 12.5 | 12.5 |
| HAP_178 | 47 | 76.6 | 12.8 | 10.6 |
| HAP_186 | 24 | 83.4 | 8.3 | 8.3 |
| HAP_227 | 20 | 90 | 0 | 10 |
| HAP_237 | 20 | 80 | 20 | 0 |
| HAP_255 | 29 | 79 | 14 | 7 |
| HAP_317 | 20 | 90 | 0 | 10 |
| HAP_398 | 20 | 90 | 0 | 10 |
|  |  |  |  |  |

| **Denovo2977**  *(Mollicutes)*  Total number of sequences: 3,480  Total number of haplotypes: 422 | | | | |
| --- | --- | --- | --- | --- |
| **Haplotypes** | | **Percentages** | | |
| Name | Number of sequences | Rainbow | TAG | Logatchev |
| HAP_4 | 2,640 | 28.4 | 69.1 | 2.5 |
| HAP_14 | 37 | 46.0 | 54.0 | 0.0 |
| HAP_34 | 20 | 100.0 | 0.0 | 0.0 |
| **Denovo2874**  *(Epsilonproteobacteria)*  Total number of sequences: 863  Total number of haplotypes: 363 | | | | |
| Name | Number of sequences | Rainbow | TAG | Logatchev |
| HAP_2 | 285 | 35.1 | 59.3 | 5.6 |
| HAP_4 | 84 | 3.6 | 81.0 | 15.4 |
| **Denovo5619**  *(Epsilonproteobacteria)*  Total number of sequences: 1,175  Total number of haplotypes: 378 | | | | |
| Name | Number of sequences | Rainbow | TAG | Logatchev |
| HAP_1 | 296 | 13.2 | 50.3 | 36.5 |
| HAP_3 | 408 | 11.3 | 49.2 | 39.5 |
| **Denovo529**  *(Epsilonproteobacteria)*  Total number of sequences: 1,690  Total number of haplotypes: 437 | | | | |
| Name | Number of sequences | Rainbow | TAG | Logatchev |
| HAP_5 | 7,18 | 0.5 | 34.5 | 65.0 |
| HAP_6 | 235 | 21.7 | 0 | 78.3 |
| HAP_133 | 69 | 95.6 | 2.9 | 1.5 |
| **Denovo8992**  *(Epsilonproteobacteria)*  Total number of sequences: 4,976  Total number of haplotypes: 521 | | | | |
| Name | Number of sequences | Rainbow | TAG | Logatchev |
| HAP_3 | 3,090 | 1.3 | 16 | 82.7 |
| HAP_15 | 258 | 0 | 0.4 | 99.6 |
| HAP_37 | 360 | 2.5 | 82 | 15.5 |
| HAP_66 | 33 | 0 | 3.1 | 96.9 |
| HAP_113 | 195 | 41.1 | 54.8 | 4.1 |
| **Denovo10556**  *(Epsilonproteobacteria)*  Total number of sequences: 6,543  Total number of haplotypes: 1,043 | | | | |
| Name | Number of sequences | Rainbow | TAG | Logatchev |
| HAP_1 | 662 | 0.3 | 3.5 | 96.2 |
| HAP_4 | 102 | 89.2 | 7.8 | 3 |
| HAP_5 | 47 | 17.0 | 6.4 | 76.6 |
| HAP_6 | 3,477 | 0 | 99.9 | 0.1 |
|  |  |  |  |  |
